# Tankyrases Promote Homologous Recombination and Check Point Activation in Response to DSBs

**DOI:** 10.1371/journal.pgen.1005791

**Published:** 2016-02-04

**Authors:** Zita Nagy, Alkmini Kalousi, Audrey Furst, Marc Koch, Benoit Fischer, Evi Soutoglou

**Affiliations:** 1 Institut de Génétique et de Biologie Moléculaire et Cellulaire (IGBMC), Illkirch, France; 2 Institut National de la Santé et de la Recherche Médicale (INSERM), U964, Illkirch, France; 3 Centre National de Recherche Scientifique (CNRS), UMR7104, Illkirch, France; 4 Université de Strasbourg, Illkirch, France; The University of North Carolina at Chapel Hill, UNITED STATES

## Abstract

DNA lesions are sensed by a network of proteins that trigger the DNA damage response (DDR), a signaling cascade that acts to delay cell cycle progression and initiate DNA repair. The Mediator of DNA damage Checkpoint protein 1 (MDC1) is essential for spreading of the DDR signaling on chromatin surrounding Double Strand Breaks (DSBs) by acting as a scaffold for PI3K kinases and for ubiquitin ligases. MDC1 also plays a role both in Non-Homologous End Joining (NHEJ) and Homologous Recombination (HR) repair pathways. Here we identify two novel binding partners of MDC1, the poly (ADP-ribose) Polymerases (PARPs) TNKS1 and 2. We find that TNKSs are recruited to DNA lesions by MDC1 and regulate DNA end resection and BRCA1A complex stabilization at lesions leading to efficient DSB repair by HR and proper checkpoint activation.

## Author Summary

MDC1 recruit Tankyrases to DNA lesions to regulate homologous recombination and to control check-point activation.

## Introduction

Maintenance of genome integrity is critical for both normal cellular functions and for suppressing mutagenic events that may lead to cancer [1] [2]. DNA damage can occur due to environmental agents such as UV light or irradiation, and endogenous sources such as oxidative by-products of cellular metabolism or stalled replication forks [2]. To prevent irreversible mutations that can occur throughout the life span of an organism, multiple repair systems have emerged during evolution [3].

Breaks that affect both DNA strands (Double Strand breaks, DSBs) are among the most lethal lesions as they can lead to the discontinuity of genetic information and chromosomal aberrations [2]. DSBs are repaired by two main pathways: Non Homologous End Joining (NHEJ) and Homologous Recombination (HR) [4]. NHEJ is used by cells to join broken ends by simple religation and although this pathway is active throughout the cell cycle, it mainly occurs during G1 [5]. The NHEJ pathway is often error prone and can drive chromosome translocations by joining distal DSBs from different parts of the genome [6]. HR functions predominantly when pairing of sister chromatids occurs during S/G2 and takes advantage of the information encoded by the homologous template to eliminate the DSB in an error-free manner [7]. During HR, DNA is processed to generate single stranded ends that are coated by RPA and subsequently by RAD51. These nucleoprotein filaments are then prone to invade the homologous strand so that subsequent repair can take place [7, 8].

Cells respond to DNA damage by initiating a signaling cascade, called the DNA damage response (DDR), which leads to the activation of cell cycle checkpoints arresting the cell cycle and allowing the cell to repair the damage before division [9]. The DDR is initiated by the recruitment and extensive spreading of DDR proteins around the lesions that results in the formation of discrete foci [10]. A key stimulator of DDR spreading is the mediator of DNA damage checkpoint protein 1 (MDC1), which guides the perpetuation of the phosphatidylinositol 3-kinase (PI3K)–ataxia telangiectasia mutated (ATM) signaling pathway as well as the spreading of ubiquitination and subsequent recruitment of checkpoint mediators such as 53BP1 and BRCA1 [11, 12]. BRCA1 is considered to be a master regulator of genomic integrity contributing to efficient repair of DSBs by HR, to DDR and to check-point activation [12].

Although it has been the focus of many studies, our knowledge of the 2089 amino acid (aa) long human MDC1 protein is not exhaustive. MDC1 was reported to directly interact with different DDR factors via its separate domains [13]. The Forkhead Associated Domain (FHA) of MDC1 was shown to be in contact with ATM, Chk2 and Rad51. The MDC1 Ser-Asp-Thr (SDT) repeats interact with the MRE11-RAD50-NBS1 (MRN) complex, while the RNF8 Binding Domain (RBM) recruits the RNF8 ubiquitin ligase to MDC1-bound sites ([13] and references therein). Furthermore, the BRCA1 C-terminal (BRCTs) repeats in the hMDC1 C-terminal domain were crystallized and shown to directly bind γ-H2AX [14]. Besides its major role as a platform for DDR signaling, MDC1 was also shown to play primordial roles in NHEJ and HR [15] [16] [17]. How one protein can fulfill these rather different roles is still an open question. Aiming bringing new knowledge concerning this point, we set forward to identify potential partners of MDC1.

We identified two novel MDC1 interacting partners, the poly-ADP-ribose polymerases (PARPs) Tankyrase 1 and 2 (TNKS1/2). We show that Tankyrases associate with DNA lesions in an MDC1-dependent fashion. Our data highlight the role of TNKSs in stabilizing the BRCA1-CtIP and BRCA1A complexes at DSBs and playing roles in HR and G2/M checkpoint activation.

## Results

### TNKS1/2 interacts with MDC1 through two binding motifs

**Fig 1:**
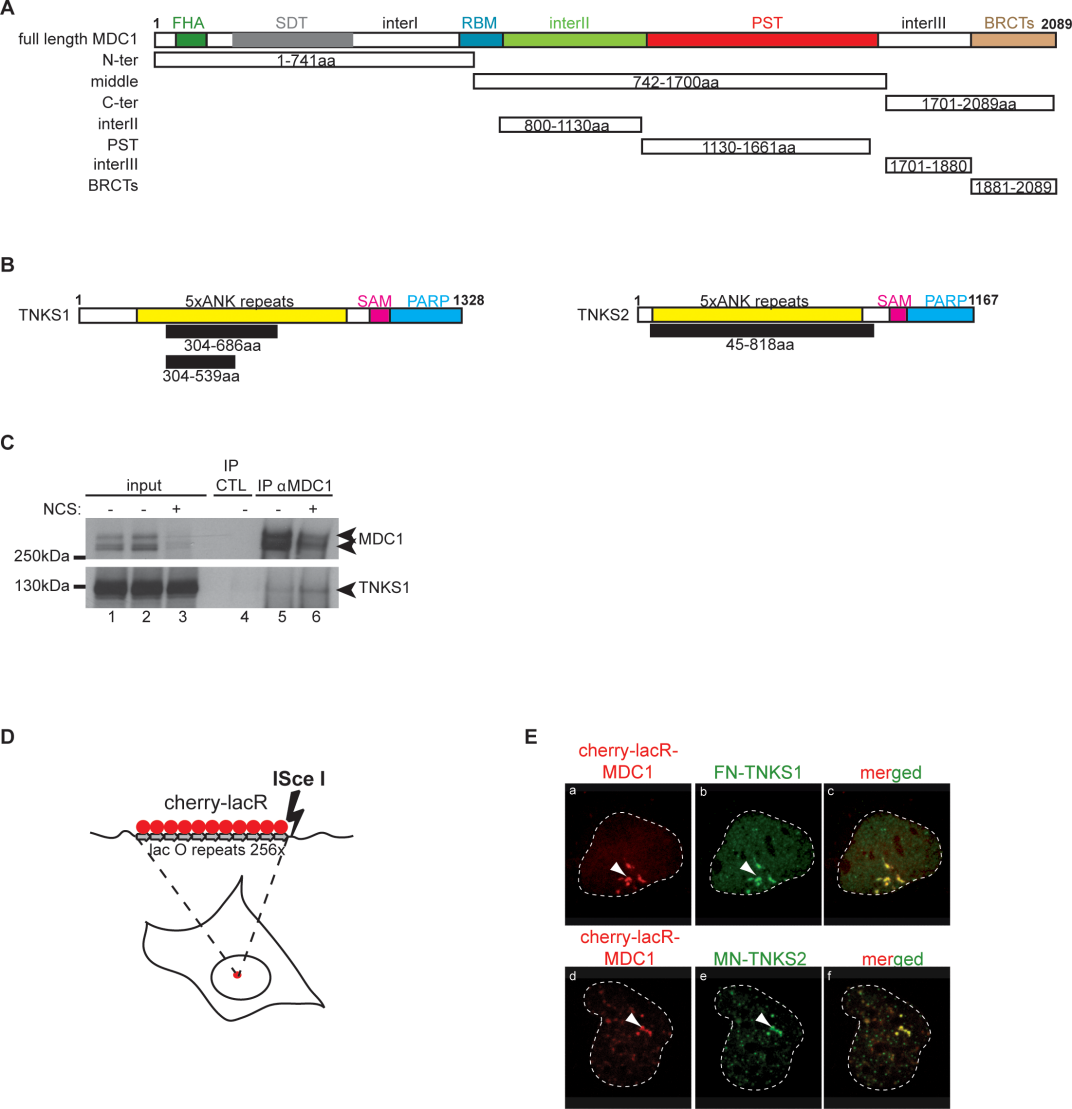
MDC1 interacts with Tankyrase 1 and Tankyrase 2. **(A)** Schematic representation of MDC1 and its domains. The different deletion constructs used in our study are depicted below the full-length protein. The domains marked are: Forkhead associated domain (FHA), SDT-repeats, RNF8 Binding motif (RBM), PST repeats, BRCA1 C-terminal domains (BRCTs). We also placed three regions with so far unknown functions, named”inter I-II-III”. **(B)** Schematic representation of the two human Tankyrase proteins with their domains; the ankyrin repeats (ANK), the sterile alpha motif (SAM) and the PARP enzymatic domain (PARP). The clones obtained in our yeast-two-hybrid screen are depicted in black below the full-length proteins. **(C)** Endogenous MDC1 and TNKS1 interact *in vivo*. HEK 293T cells were treated or not with 100 ng/ml NCS and the cell extracts subjected to immunoprecipitation using an anti-MDC1 antibody. TNKS1 is detected in the immunoprecipitate and the interaction is enhanced after DSB induction. **(D)** Schematic representation of the tethering system used in our study. **(E)** Both TNKS1 and TNKS2 interact with chromatin bound MDC1 *in vivo*. Full length MDC1 was tethered to chromatin in U2OS17 cells (for the system used see panel D) while cells were co-transfected with tagged versions of either TNKS1 or TNKS2. Colocalization of FN-TNKS1 or MN-TNKS2 with mCherry-lacR-MDC1 is detected by immunofluorescence staining.

To get insights into the mechanism of action of MDC1, we searched for novel interacting partners. We conducted a yeast two-hybrid screen of a human placenta cDNA library using MDC1 as bait (Fig 1A). Interestingly, two poly-ADP-ribose polymerases (PARPs), TNKS1 (PARP5a) and TNKS2 (PARP5b), were identified as proteins that interacted with the MDC1 “middle” domain (Fig 1B). The clones identified in this screen harbored the ankyrin repeats of Tankyrases, a domain known for establishing protein-protein interactions (Fig 1B) [18, 19]. For detailed results of the yeast two-hybrid screen see S1 Fig.

To verify this interaction in human cells with the endogenous proteins, we performed co-immunoprecipitation experiments followed by Western Blot. As shown in Fig 1C, endogenous MDC1 interacts with TNKS1 and this association increases in the presence of the radiomimetic drug Neocarzinostatin (NCS). The decrease in the levels of MDC1 upon DNA damage is possibly due to the proteolytic degradation of MDC1, as shown in [20] (see Fig 1C lane 3). To further prove this interaction, we utilized our *in vivo* lacO-LacR targeting system (Fig 1D) that we have previously used to dissect the hierarchy of DNA repair factor recruitment at DDR foci [21]. Since the antibody recognizes mainly TNKS1, we expressed tagged versions of TNKS1 or TNKS2 in the cells. Full-length mCherry-lacR-MDC1 efficiently recruited both flag-tagged TNKS1 and myc-tagged TNKS2 to the LacO array (Fig 1E).

**Fig 2:**
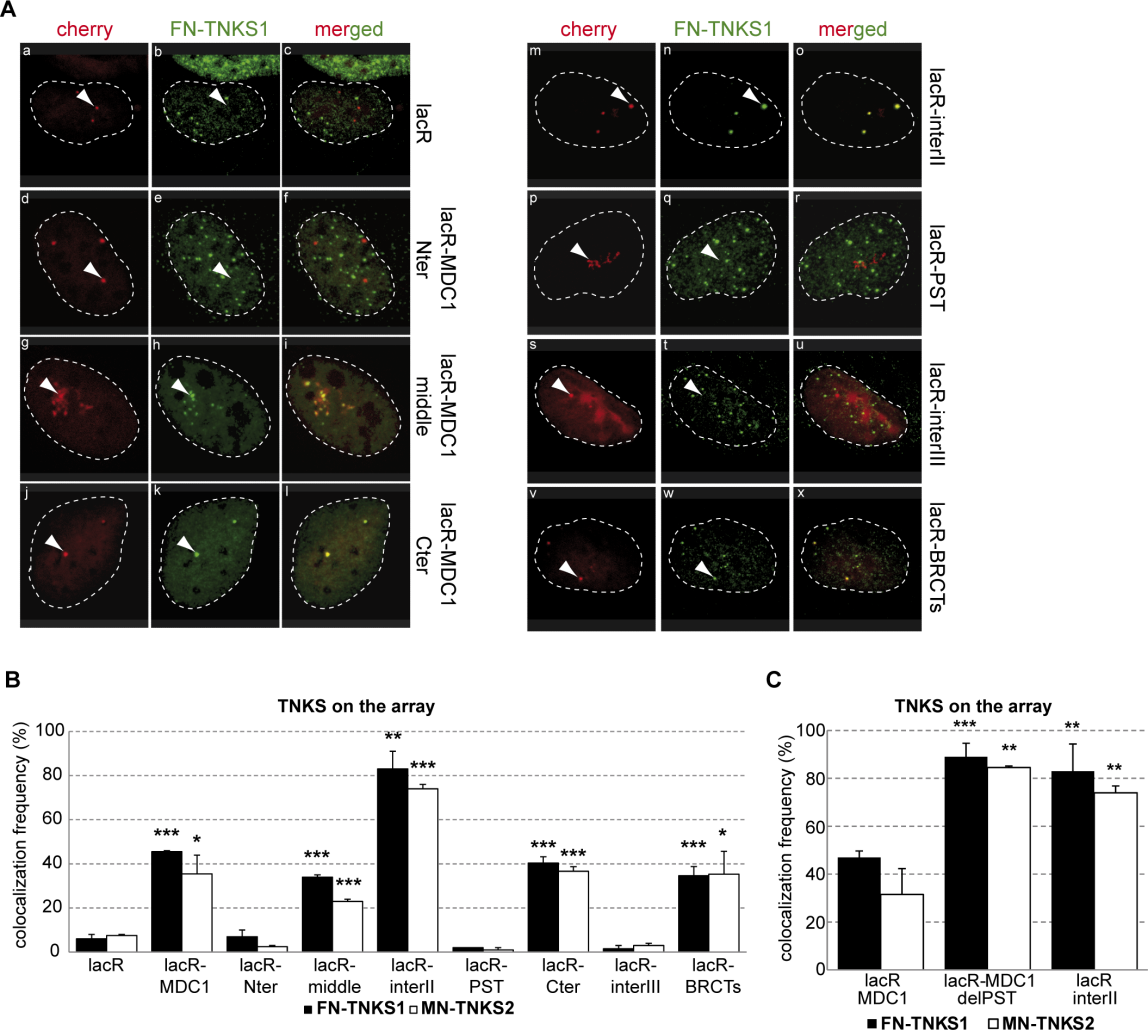
Mapping of the Tankyrase interacting domain of MDC1. **(A)** Confocal microscopy images of U2OS17 cells transfected with different mCherry-lacR-MDC1 domain constructs and flag tagged TNKS1 (FN-TNKS1). The contour of nuclei is shown. Colocalization of the two proteins can be observed in the merged images. **(B)** Quantification of the colocalization frequency of flag-TNKS1 (FN-TNKS1) or myc-TNKS2 (MN-TNKS2) and the different tethered MDC1 domains. Values represent mean ± SD from three independent experiments (N = 100 cells in each experiment). Statistical significance in all relevant Figs was calculated using the t-test (P<0,05 *, P<0,01 **, P<0,001 ***) **(C)** The PST repeats of MDC1 regulate the interaction between Tankyrases and MDC1. U2OS17 cells were transfected with different constructs expressing cherry-lacR fused versions of MDC1 (marked on the X axis) and Tankyrase (TNKS1 or TNKS2). % of cells having Tankyrase on the array was determined as for panel (B).

To precisely map the MDC1 domain that recruits TNKSs, we generated deletion mutants of MDC1 fused to mCherry-lacR (Fig 1A) and expressed them in U2OS17 cells (harboring the lacO repeats, Fig 1D). We found that the poorly characterized central region (742-1700aa, Fig 1A) called hereafter the “middle” domain is sufficient to recruit both TNKSs (Fig 2A, panels g-i and Fig 2B). TNKSs also interacted with equal efficiency with the MDC1 C-terminal region (panels j-l in Fig 2A) but not with the MDC1 N-terminal domain (panels d-f in Fig 2A, see Fig 2B for quantifications).

To map more precisely the TNKS Binding Domains (TBDs) of MDC1, we continued our analysis by narrowing down the MDC1 regions that yield positive signals (for the constructs see Fig 1A). Further deletion analysis of this “middle” domain showed that although the Proline-Serine-Threonine (PST) region (1130-1661aa) does not interact with TNKSs (panel p-r in Fig 2A), the domain between the known RNF8 binding domain (RBM) and PST, referred here as “interII” (800-1130aa) (Fig 1A), yielded in very efficient colocalization with TNKS1 or 2 (panels m-o in Fig 2A, for quantifications see Fig 2B). Also, an interaction was detected between the BRCT domain of MDC1 (1881-2089aa) and TNKS1/2 (panels v-x in Fig 2A, for quantifications see Fig 2B).

**Fig 3:**
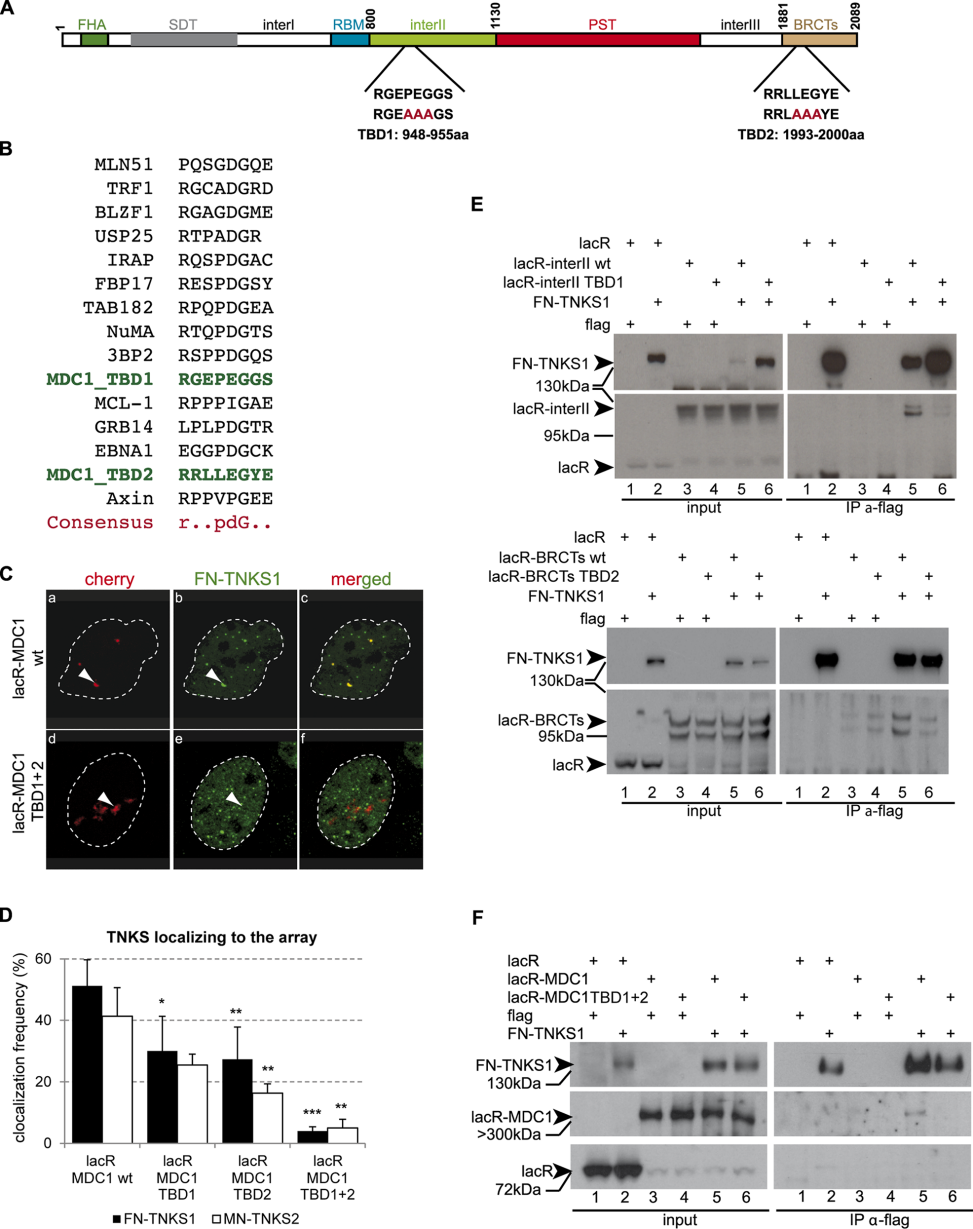
The two Tankyrase binding domains of MDC1 act cooperatively. **(A**) Schematic representation of MDC1 and the two predicted TBDs with their sequences highlighted. Directed mutagenesis was conducted to replace 3 amino acids of each TBD to Alanine (shown in red capital letters). **(B)** Multiple alignment of known TBDs from the literature and the consensus sequence. The two predicted TBDs of MDC1 are highlighted in green. **(C)** Immunofluorescence staining of U2OS17 cells co-transfected with FN-TNKS1 and mCherry-lacR-MDC1 wild type or mCherry-lacR-MDC1 TBD mutant. The mutant version does not colocalize any more with TNKS. **(D)** Quantification of the colocalization frequency of full-length mCherry-lacR-MDC1 (wt or TBD mutants) and TNKS1/2. Disruption of the two TBDs by directed mutagenesis leads to the loss of colocalization. Values represent mean ± SD from three independent experiments (N = 100 cells in each experiment). **(E)** *In vivo* interaction of TNKS1 and the MDC1 “interII” or “BRCTs” domain detected by co-immunoprecipitation. HEK 293T cells were co-transfected with either mCherry-lacR (lanes 1,2), mCherry-lacR-interII (lanes 3–6, upper panel) or mCherry-lacR-BRCTs (lanes 3–6, bottom panel) and FN-TNKS1 (in lanes 2, 5, 6). TBD mutant versions of mCherry-lacR-interII or mCherry-lacR-BRCTs (lanes 4, 6 on upper and bottom panel, respectively) were also included in the analysis. Immunoprecipitation was performed with the M2 flag antibody and the immunoblot was developed with an anti-lacR antibody revealing an interaction between FN-TNKS1 and lacR-interII or lacR-BRCT. Flag antibody was used to detect the presence of FN-TNKS1. Arrowheads show the specific bands. **(F)** *In vivo* interaction of TNKS1 and MDC1 requires intact TBD domains. HEK 293T cells were transfected with mCherry-lacR (lanes 1, 2) or mCherry-lacR-MDC1 (lanes 3–6) and FN-TNKS1 (lanes 2, 5, 6). MDC1 was mutated on its two TBDs in lanes 4 and 6. Cell extracts were subjected to anti-flag immunoprecipitaton and analysed by Western Blotting. Only the wild type MDC1 co-precipitates with FN-TNKS (IP lane 5).

The finding that the colocalization frequency between tethered lacR-interII-MDC1 and TNKSs is higher than that of the full length MDC1 (MDC1 FL) or the “middle” region, points to an inhibitory role of the PST domain in this interaction. In line with this observation, deletion of the PST domain in the context of MDC1 FL increased the colocalization with TNKSs at the lacO array after tethering (Fig 2C). A comparison of the amino acid sequences of the “interII” and “BRCTs” domains and that of known TBDs from the literature [18] allowed us to map the two potential TBDs within the MDC1 sequence at positions 948-955aa (“interII” domain) and 1993-2000aa (“BRCTs” domain) (Fig 3A and 3B). Site directed mutagenesis of three amino acids of the eight present in the binding motifs to alanine (Fig 3A and 3B) showed that disrupting one TBD leads to decreased interaction, but mutations in both TBDs are required for complete loss of the MDC1 and TNKS colocalization signal (Fig 3C and 3D). To confirm that the association of TNKSs with MDC1 depends on the above-described TBDs, we performed co-immunoprecipitation studies on extracts overexpressing mCherry-lacR-interII or mCherry-lacR-BRCT harboring or not the TBD mutations as well as the full-length mCherry-lacR-MDC1 (wt or TBD mutant) with FN-TNKS1 (Fig 3E and 3F). We indeed found that mutations at the TBDs diminish the interaction with TNKS1 FL or deletion mutants (Fig 3E and 3F).

Overall, these observations establish the existence of two TBDs within the MDC1 sequence that ensure the interaction with Tankyrases *in vivo*.

### TNKS1/2 are recruited to DNA breaks in an MDC1-dependent manner

**Fig 4:**
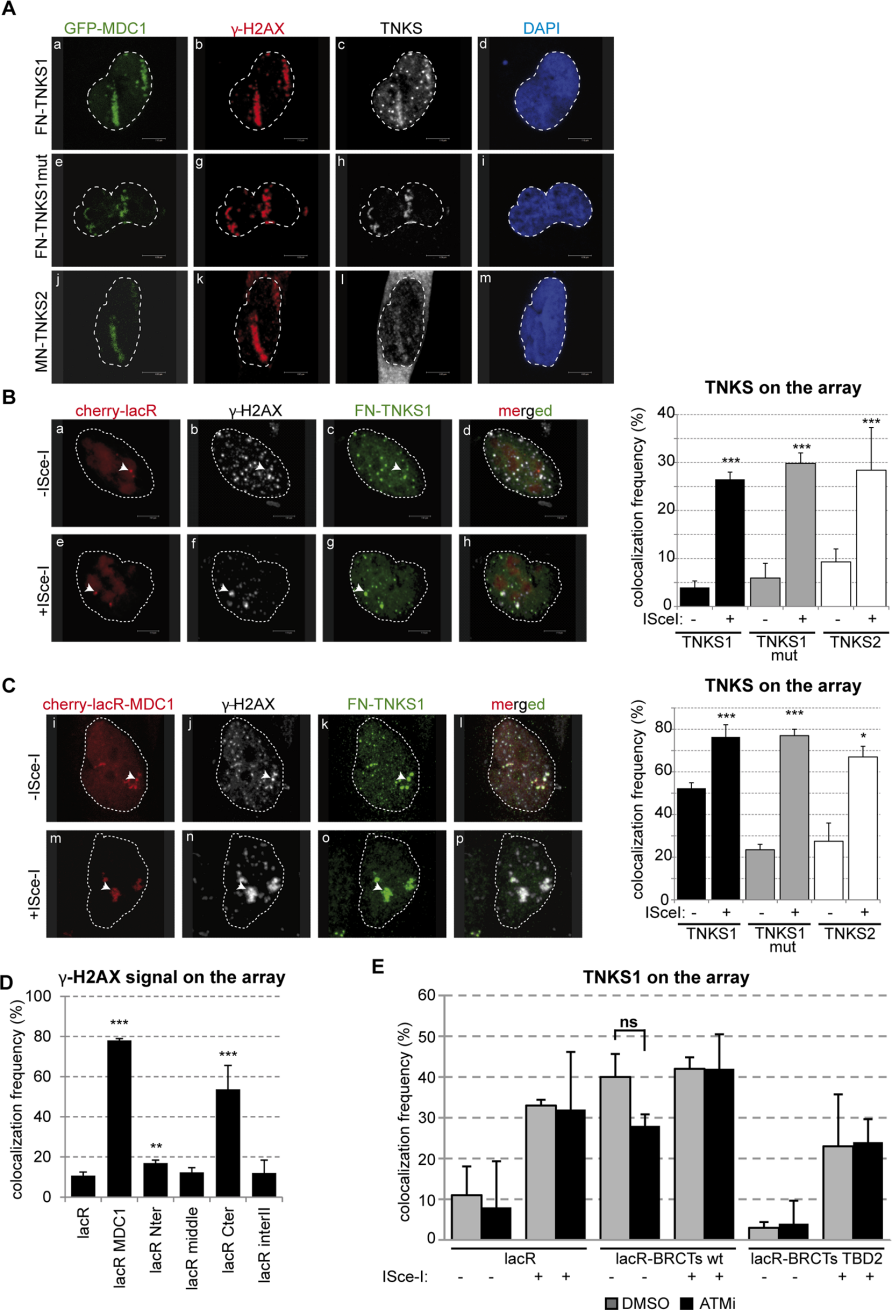
Tankyrase1/2 localizes to DSBs. **(A)** Confocal microscopy pictures of U2OS cells co-transfected with GFP-MDC1 and FN-TNKS1 or MN-TNKS2 after laser-induced DNA damage. Cells were fixed and stained for γ-H2AX, flag or myc using the appropriate antibodies. **(B)** Confocal microscopy images of U2OS17 cells co-transfected with mCherry-lacR and FN-TNKS1. The cells were also transfected with an ISce-I expressing plasmid in panels e-h. TNKS localizes to pure DSBs as visualized on panels e-h. The quantification of TNKS localization frequency to DSBs is shown on the right, values obtained from three independent experiments are shown with SD (N = 100). **(C)** Confocal microscopy pictures of U2OS17 cells co-transfected with mCherry-lacR-MDC1 and FN-TNKS1. An ISce-I expressing plasmid was also transfected on panels m-p. Values are calculated as on panel (B). **(D)** Targeting of FL MDC1 to chromatin leads to DDR activation. U2OS17 cells were transfected with the indicated constructs, and the % of cells harboring γ-H2AX signal on the array was quantified. Results of three independent experiments are shown with SD (N = 100). **(E)** ATM mediated phosphorylation events do not affect the interaction between TNKS and the BRCTs of MDC1. U2OS17 cells were transfected with the indicated plasmids in the presence or absence of ATMi. Immunofluorescence staining was performed and the % of cells having FN-TNKS1 signal on the array quantified in three independent experiments (N = 100). Average values are shown with SD.

The above results concerning the interaction between MDC1 and TNKS1/2 suggest that TNKSs might localize to sites of DNA damage. To investigate this possibility, we first inflicted DNA damage in U2OS cells using an 800nm multiphoton laser. Efficient damage was visualized by the recruitment of GFP-MDC1 and the phosphorylation of H2AX γ-H2AX) at the laser stripes (Fig 4A). Both TNKS1 and TNKS2 as well as a PARP dead mutant version of TNKS1 were visualized at the sites of DNA damage that colocalized with the above DDR markers. (panels c, h and l in Fig 4A).

We next sought to follow the potential recruitment of Tankyrases at site-specific DSBs by taking advantage of the presence of a unique ISce-I homing endonuclease recognition site that lies adjacent to the lacO repeats in U2OS17 cells (Fig 1D). Notably, we observed colocalization of the mCherry-lacR spot with TNKSs only in cells expressing the ISce-I endonuclease (Fig 4B) suggesting that TNKSs are recruited to “pure” DBSs. Subsequent quantification of TNKS colocalization with the ISce-I-induced breaks showed that TNKSs are recruited to DNA lesions in a fraction of cells (around 30%, Fig 4B). Notably, the PARP dead mutant version of TNKS1 was recruited as efficiently as the wild type protein, suggesting that the PARP activity of TNKSs is not required for their localization to DNA damage sites (Fig 4B, graph on the right, see also S2 Fig). Since the tethering of MDC1 to the lacO array is sufficient to recruit TNKSs (see Figs 1E and 2B), we next assessed whether induction of ISce-I breaks next to MDC1-bound chromatin further increased the occupancy of the lacO array by TNKSs. Indeed, upon ISce-I break induction the MDC1-TNKS colocalization frequency was higher and the signal intensity on the array was stronger (panels n-p and graph in Fig 4C).

As MDC1 tethering was previously shown to trigger DDR [21], we tested if TNKS loading requires this activity of the MDC1. Our results show that the DDR depends on the C-terminal end of MDC1 (Fig 4D), while the interII domain that recruits most efficiently TNKSs to the chromatin (see Fig 2B) does not activate such a response (Fig 4D). To further test whether the interaction of TNKSs with the BRCT domain depends on its capacity to activate DDR, we inhibited DDR induced by BRCT after tethering using the ATM inhibitor. We find no effect on the recruitment efficiency of TNKS1 on tethered BRCT at the array (Fig 4E). Thus, the abilities of MDC1 to trigger damage response and to load TNKSs to the chromatin are independent.

**Fig 5:**
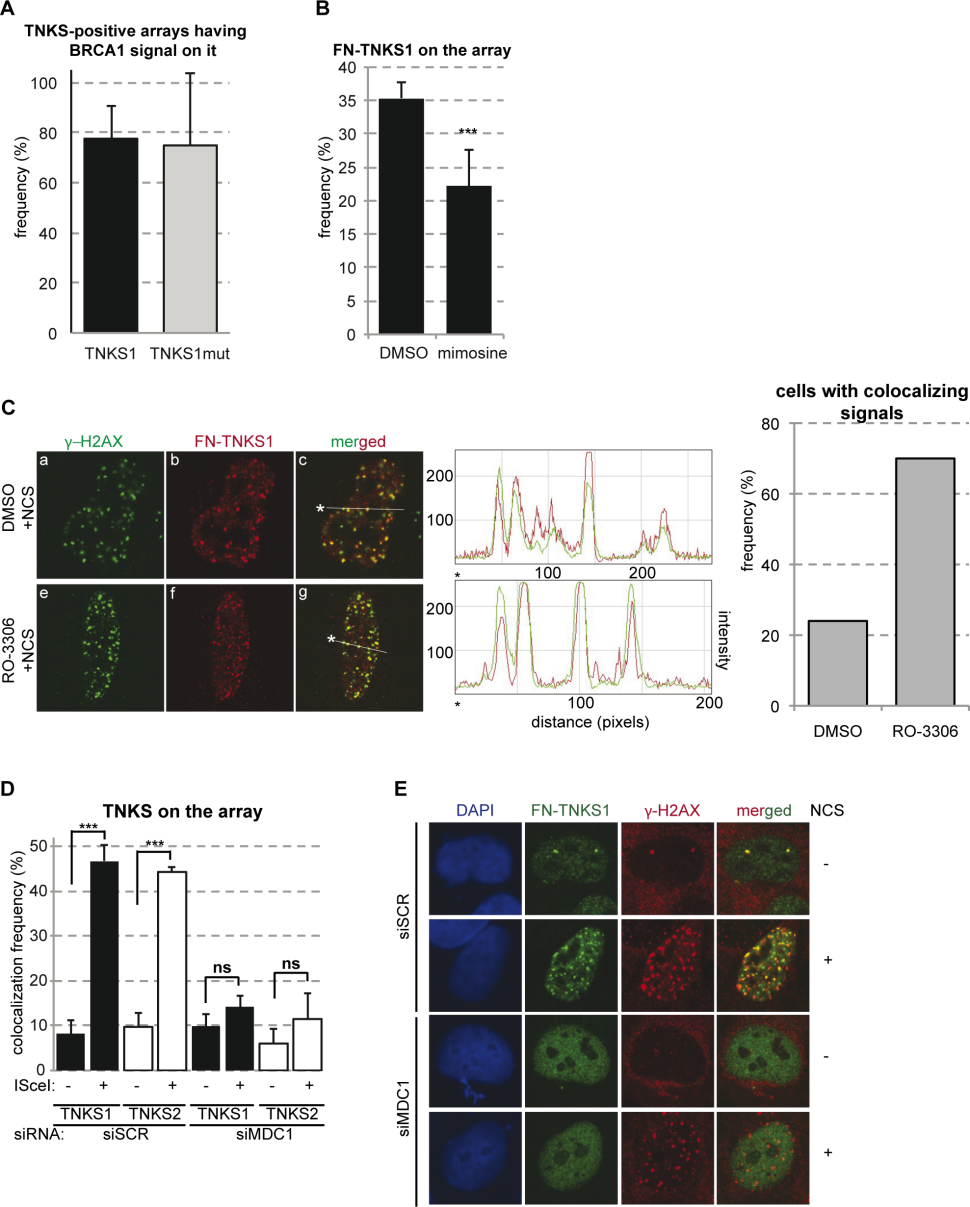
TNKSs are recruited to DSBs in a cell cycle dependent manner. **(A)** Quantification of the frequency of colocalization of TNKS and BRCA1 at pure DSBs *in vivo*. U2OS17 cells transfected with mCherry-lacR, FN-TNKS1 and ISce-I, and were co-stained with antibodies against flag and BRCA1. Percent of cells having TNKS1 signal at the lacO array also positive for BRCA1 was determined. Results from three independent experiments are shown with SD (N = 30). **(B)** Quantification of the frequency of TNKS1 recruitment to pure DBSs in control or G1 blocked cells. U2OS17 cells were transfected with mCherry-lacR, FN-TNKS1 and ISce-I. 20hours before fixation, cells were treated with mimosine to block the cell cycle in G1. Percent of cells with FN-TNKS1 signal on the lacO array was determined. Results from three independent experiments are shown with SD (N = 100). **(C)** TNKS1 is recruited to DSBs *in vivo* in a subset of cells. U2OS cells were transfected with FN-TNKS1, treated with NCS and immunostained with antibodies against flag and γ-H2AX. Cells were blocked in G2 phase by the CDK1 inhibitor RO-3306 on panels e-g. Frequency of cells showing a colocalizing signal for γ-H2AX and FN-TNKS increased from 24% (asynchronous cells) to 70% (G2 cells) as shown on the graph on the right. Colocalization was analyzed by plotting the intensity of the red/green signal against the distance along the white line on the merged image. The starting point is marked by a star. **(D)** MDC1 is required for TNKS recruitment to pure DSBs *in vivo*. U2OS17 cells were transfected with control or MDC1 targeted siRNAs. 24hours after siRNA treatment, cells were co-transfected with mCherry-lacR, FN-TNKS1 or MN-TNKS2 and ISce-I. The frequency of cells having TNKS1/2 signal on the array was determined, results of three independent experiments are shown with SD (N = 100). **(E)** MDC1 is required for TNKS foci formation. U2OS cells were transfected with control or MDC1-directed siRNAs. 48 hours later the cells were transfected with FN-TNKS1 and 24 hours later treated with 100ng/ml NCS. 6 hours after treatment cells were stained with antibodies against γ-H2AX and FN-TNKS. Representative confocal microscopy pictures are shown.

The frequency of recruitment of TNKSs to pure DSBs (~30%, see Fig 4B) suggests that a cell cycle-specific recruitment occurred. To test this idea, we quantified the percentage of TNKS and BRCA1 colocalization in ISce-I-inflicted breaks. We found that the vast majority of ISce-I breaks marked with TNKSs were also marked with BRCA1 (~80%, Fig 5A), which further points to a preferential recruitment of TNKSs to DNA damage in the S and G2 phases of the cell cycle. In line with this observation, the recruitment of TNKS1 at the lacO/ISce-I locus was reduced in cells arrested in G1/S by mimosine (Fig 5B).

The colocalization of TNKSs with naturally occuring γ-H2AX foci (panel b and c in Fig 4B) further reinforces the hypothesis about their presence at naturally occurring DNA lesions. Moreover, FN-TNKS1 exerted colocalization with γ-H2AX foci induced by NCS (Fig 5C). This colocalization increased significantly in the case of FN-TNKS1 in cells arrested in G2 by RO-3306 (Fig 5C). In line with this observation, the expression and the nuclear localization especially of TNKS1 seems to increase in S and G2 phases of the cell cycle (S3A and S3B Fig). To establish if TNKS recruitment to breaks requires MDC1, we quantified the frequency of TNKS1/2 localization to the lacO array in the presence or absence of ISce-I breaks in control and MDC1 depleted cells. Depletion of MDC1 significantly affected the recruitment of TNKSs to ISce-I-induced breaks (Fig 5D), and hampered efficient TNKS1 foci formation after NCS treatment (Fig 5E). On the other hand, downregulation of TNKSs had no effect on MDC1 recruitment to single DSBs in U2OS17 cells (S4A Fig) neither on MDC1 foci formation after DSB induction with the radiomimetic drug NCS (S4B Fig). Efficient depletion of MDC1 and TNKSs was assessed by RT-PCR and Western blot analysis or immunofluorescence staining (S5A and S5B Fig). Our findings thus provide evidence for MDC1-dependent recruitment of Tankyrases to DSBs *in vivo* as part of the early response to DNA damage.

### Tankyrases are required for efficient HR

**Fig 6:**
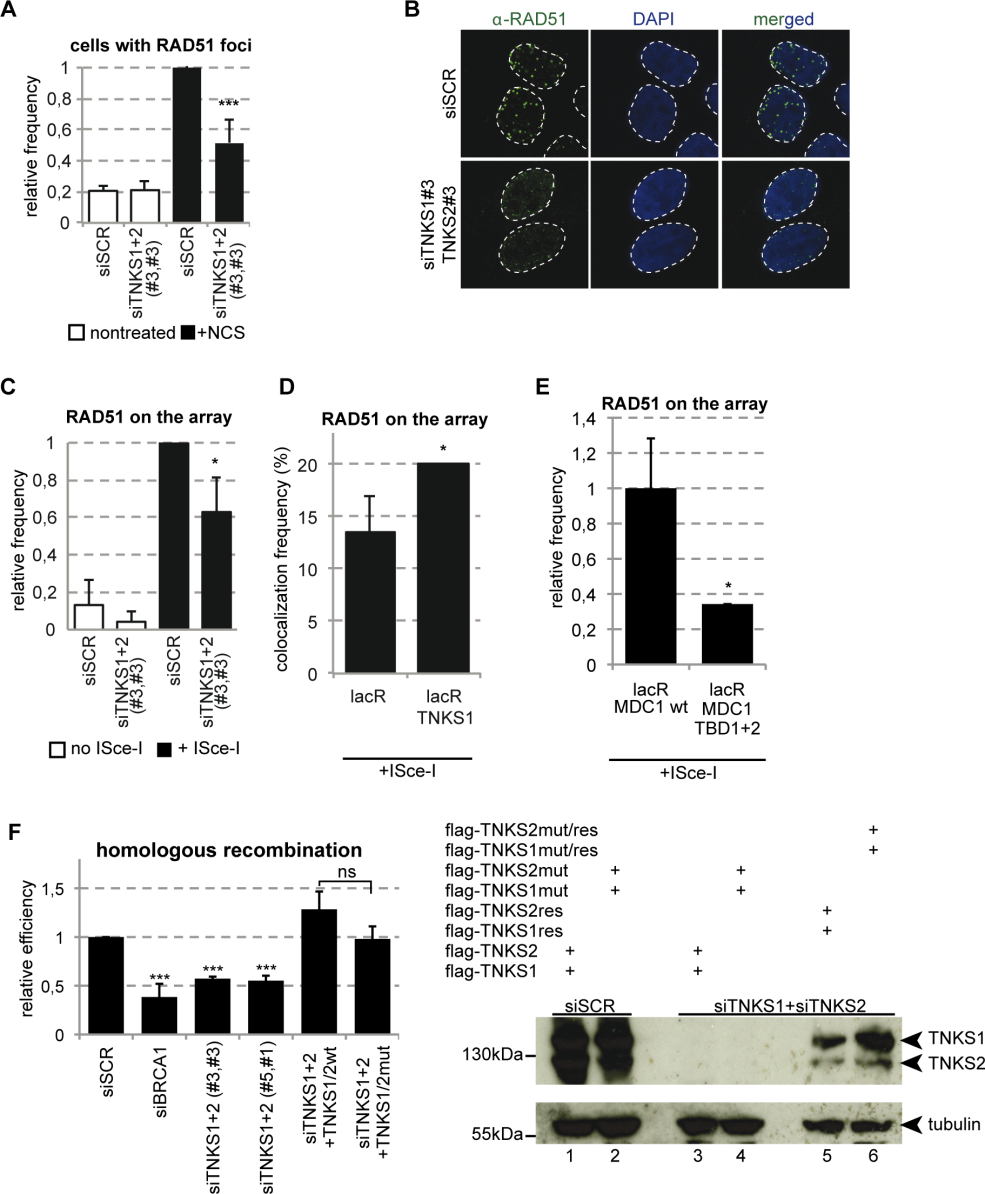
Tankyrase1/2 plays a role in RAD51 recruitment to DSBs and efficient HR. **(A)** Cells depleted of TNKS1/2 show defective foci formation of RAD51. U2OS cells were treated with NCS and fixed 6 hours later. Frequency of RAD51 foci positive cells was determined in three independent experiments (N = 100). Results are shown as relative frequencies compared to control. **(B)** Representative confocal microscopy images corresponding to panel (A). **(C)** Quantification of RAD51 on pure DSBs in U2OS17 cells reveals a drop in siTNKS conditions. U2OS17 cells were transfected with the indicated siRNAs, and then co-transfected with mCherry-lacR and ISce-I. Cells were fixed and stained using an antibody against RAD51. Colocalization frequency of the lacR and the RAD51 signal was determined. Relative frequencies compared to the control are results of three independent experiments with SD. **(D)** Forced binding of TNKS1 to a DSB increases RAD51 recruitment efficiency. U2OS17 cells were transfected with GFP-lacR or GFP-lacR-TNKS1 and ISce-I. The percentage of cells having RAD51 signal on the array was determined after immunofluorescence staining. Results from three independent experiments are shown with SD (N = 100). **(E)** MDC1-mediated TNKS recruitment is necessary for efficient RAD51 binding to DSBs *in vivo*. U2OS17 cells were transfected with mCherry-lacR-MDC1 wt or its TBD mutant version and ISce-I. Percent of cells harboring RAD51 signal on the array was determined. Results from three independent experiments are shown with SD (N = 100). **(F)** HR efficiency is decreasing in TNKS1/2 depleted HRind cells measured in a stable cellular system [49]. Results of three independent experiments are shown with SD. N>10^5^ cells were observed by FACS for GFP expression. Western blot analysis of rescue-efficiency is shown on the right. “res” stands for siRNA rersistant version of the construct, “mut” stands for mutation in the PARP activity.

In a recent report, TNKS2 was shown to interact with RAD54 [18]. This result points to a potential role of TNKSs in DSB repair by HR via loading of HR factors at DNA lesions [22] [23]. Unfortunately, we were unable to visualize RAD54 foci after NCS treatment, and we therefore set forward to analyze the frequency of RAD51 foci in cells depleted for Tankyrases. We indeed observed a substantial drop in the number of cells forming RAD51 foci after NCS treatment (Fig 6A and 6B). A similar result was obtained in U2OS17 cells depleted for Tankyrase1/2 and transfected with ISce-I. Cells in which Tankyrases were downregulated showed a lower frequency of RAD51 accumulation at lacO/ISce-I breaks (Fig 6C). To verify if Tankyrases indeed favor the recruitment of RAD51 to pure DSBs, we tethered Tankyrase 1 fused to GFP-lacR to the chromatin and induced DSBs by ISce-I. Forced binding of TNKS1 to the lacO array resulted in increased RAD51 recruitment (Fig 6D). To investigate whether the effect of TNKSs on RAD51 foci formation depends on their interaction with MDC1, we performed rescue experiments. To this end, RAD51 foci were quantified in U2OS cells depleted for MDC1 and complemented with either wtMDC1 or the TBD mutant. As expected, depletion of MDC1 reduced RAD51 foci formation and this effect was rescued by expression of wtMDC1 but not the TBD mutant (S6A Fig). Although this result point to the importance of MDC1-TNKS interaction on RAD51 foci formation, we noticed that the MDC1 TBD mutant exerted decreased ability to form foci compared to the wt (S6B Fig). Therefore with this experiment we cannot separate the two functions. To overcome this limitation, we tethered wt and TBD mutant of MDC1 to the lacO array and assessed RAD51 recruitment after ISce-I cutting. Interestingly, tethering of the mutant leads to a significant drop in RAD51 recruitment after DSB induction with ISce-I compared to the wt MDC1 (Fig 6E).

In line with the effect of TNKSs’ depletion on RAD51 loading to lesions, Tankyrase knock down hampered the efficient repair by HR at a level comparable to our positive control, the BRCA1 knock down (Fig 6F). It is worth noting that the cell cycle profile of TNKS KD cells proved similar to control cells (S3C Fig) and a more detailed analysis for the ratio of replicating cells showed no difference compared to the siSCR control either (S3D Fig). The efficiency of BRCA1 depletion by siRNA was verified by WB (S5C Fig).

To investigate whether the role of TNKSs in HR depends on their PARP activity, we performed the same assay in cells depleted for TNKS1 and 2 and complemented with wild type TNKS1 and 2 or the combination of their PARP dead counterparts. Interestingly, wt as well as PARP dead TNKSs rescue the defect in HR, pointing to a structural role of TNKSs in DNA repair by HR that doesn't require the catalytic activity (Fig 6F). In line with this observation, inhibition of TNKSs’ PARP activity using the inhibitor XAV-939 had no effect in HR (S6C and S6D Fig).

**Fig 7:**
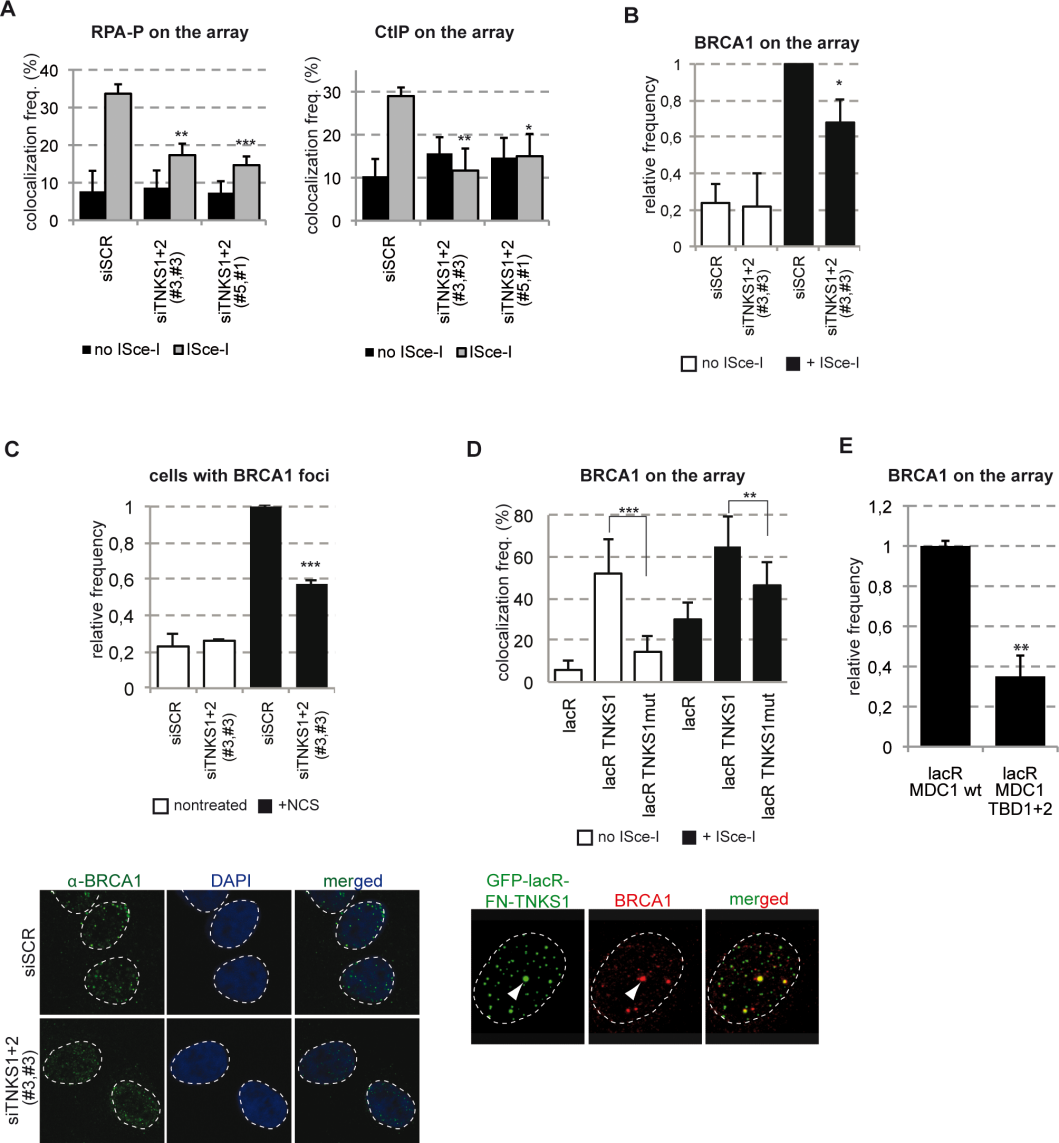
TNKSs recruit the CtIP-BRCA1 complex to chromatin. **(A)** TNKS depletion leads to defects in CtIP loading and RPA phosphorylation. U2OS19 cells were transfected with the indicated siRNAs and pure DSBs were generated by transfecting ISce-I together with mCherry-lacR. Colocalization frequency of RPA-P or CtIP with the lacO array was established in 100 cells in three independent experiments. Values represent mean ± SD. **(B)** TNKS is required for the recruitment of the BRCA1 to pure DSBs *in vivo*. U2OS17 cells were transfected with the indicated siRNAs, and then co-transfected with mCherry-lacR and ISce-I. Immunofluorescence staining with an anti-BRCA1 antibody was performed and the colocalization frequency of the lacR and BRCA1 signal quantified. Relative values compared to the control are the results of three independent experiments with SD (N = 100 cells in each condition). **(C)** TNKS depleted cells are deficient for BRCA1 foci formation. U2OS cells were transfected with siRNAs as shown and treated with NCS 48 hours later. Cells were fixed 6 hours after treatment, immunofluorescence staining detecting BRCA1 was performed. The frequency of foci-positive cells was determined in three independent experiments (N = 100). Representative confocal microscopy pictures for BRCA1 staining are shown on the bottom. **(D)** TNKS tethering to the chromatin results in BRCA1 recruitment in a partially PARP activity dependent manner. U2OS17 cells were transfected with the indicated plasmids and immunofluorescence staining was performed to establish colocalization frequencies between GFP-lacR-TNKS1 and BRCA1. Representative confocal microscopy pictures are shown on the bottom. **(E)** MDC1-mediated TNKS recruitment is necessary for efficient binding of BRCA1 to DSBs *in vivo*. U2OS17 cells were transfected with mCherry-lacR-MDC1 wt or its TBD mutant version and ISce-I. Percent of cells harboring BRCA1 signal on the array was determined. Results from three independent experiments are shown as relative frequencies compared to control with SD (N = 100).

To get further insight into the mechanism by which TNKSs regulate HR, we investigated their role in DNA end resection. To this end, we assessed two markers of resection, CtIP and the phosphorylated form of RPA (RPA-P) at ISce-I breaks in control cells and cells depleted for TNKSs. Interestingly, downregulation of TNKSs reduced substantially the signal of these markers at the LacO/IsceI locus after break induction (Fig 7A). Since CtIP was shown to act together with BRCA1, we tested the efficiency of recruitment of BRCA1 at ISce-I and NCS induced breaks in cells depleted for TNKSs. We find that downregulation of TNKSs affect the recruitment of BRCA1 to DSBs *in vivo* (Fig 7B and 7C). Moreover, TNKS1 tethering to the lacO array itself is enough to recruit BRCA1 (Fig 7D). This effect is mediated by MDC1 since tethering of the Tankyrase binding mutant form of MDC1 does not lead to the recruitment of BRCA1 as compared with the wt (Fig 7E).

Altogether our data shed light on a role of Tankyrases in HR via mediating the recruitment of the CtIP-BRCA1 complex to DSBs.

### The recruitment of the BRCA1A complex to DSBs requires Tankyrases

**Fig 8:**
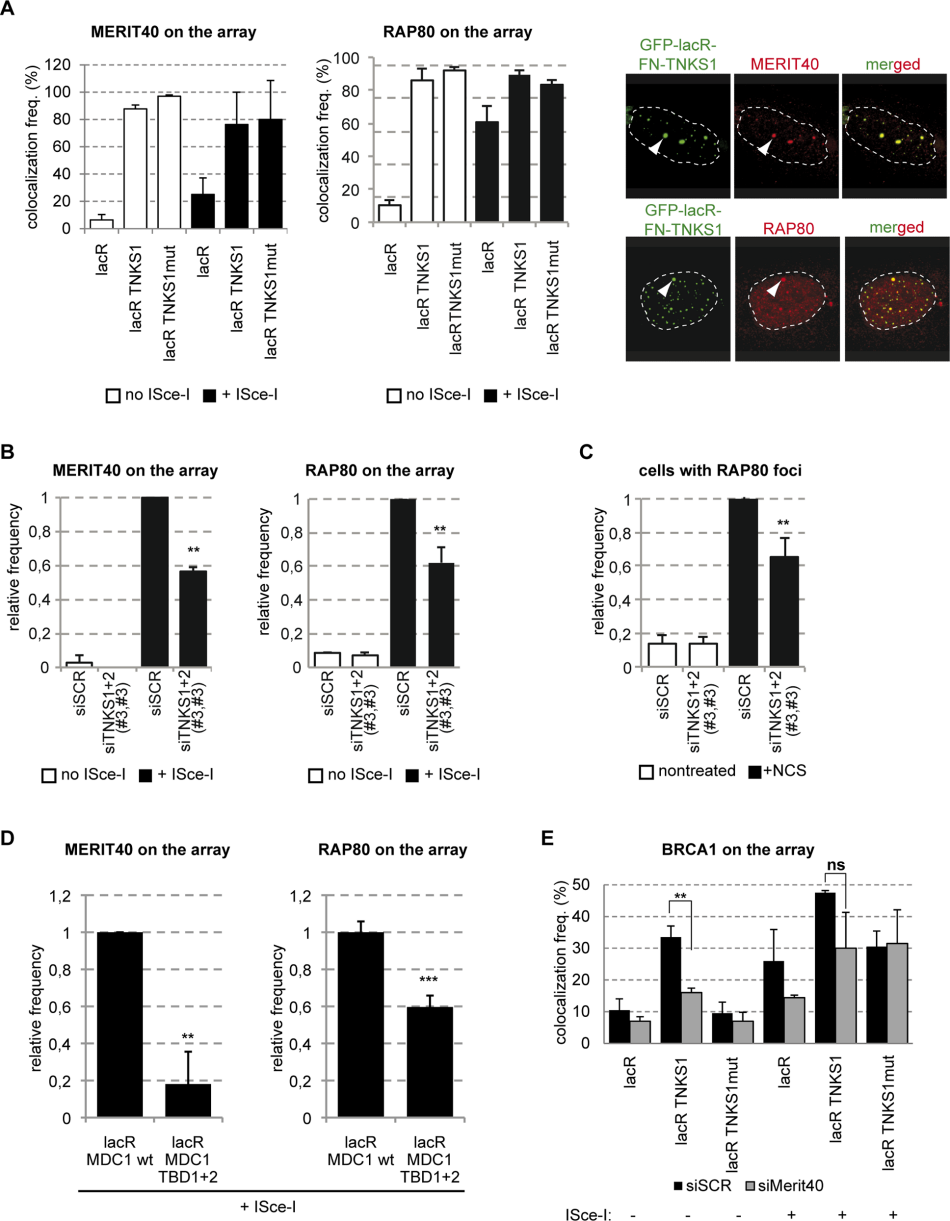
TNKS loads the BRCA1A complex on chromatin. **(A)** TNKS tethering to the lacO array leads to MERIT40 and RAP80 binding in a PARP activity independent manner even in the absence of DSBs. U2OS17 cells were transfected with GFP-lacR, GFP-lacR-TNKS1 or the PARP activity mutant version: GFP-lacR-TNKS1mut. ISce-I was co-transfected to induce pure DSBs at the lacO array. Immunofluorescence staining was performed to visualize the localization pattern of MERIT40 or RAP80 in the cells. The values represent the results of three independent experiments with SD (N = 100). On the right representative confocal microscopy pictures are shown. **(B)** The recruitment of MERIT40 and RAP80 is affected in TNKS1/2 knock down cells. U2OS17 cells were transfected with the indicated siRNAs. After depletion, cells were transfected with mCherry-lacR and ISce-I where indicated. Immunofluorescence staining was performed to visualize Merit40 or RAP80 in the cells. Relative values compared to the control are the results of three independent experiments with SD (N = 100 cells in each condition). **(C)** TNKS depleted cells are deficient for RAP80 foci formation. U2OS cells were transfected with siRNAs as shown and treated with NCS 48 hours later. The frequency of foci-positive cells was determined in three independent experiments (N = 100) and is shown as relative to the control **(D)** MDC1-mediated TNKS recruitment is necessary for efficient binding of the BRCA1 complex to DSBs *in vivo*. U2OS17 cells were transfected with mCherry-lacR-MDC1 wt or its TBD mutant version and ISce-I. Percent of cells harboring MERIT40 or RAP80 signal on the array was determined after immunofuorescence staining. Results from three independent experiments are shown with SD (N = 100) as relative frequencies compared to control. **(E)** MERIT40 stabilizes BRCA1 on the TNKS-bound chromatin. Control or MERIT40 depleted U2OS17 cells were transfected with GFP-lacR, GFP-lacR-TNKS1, GFP-lacR-TNKS1mut and ISce-I. Percent of cells harboring BRCA1 signal on the array was determined. Results from three independent experiments are shown with SD (N = 100).

Besides its interaction with RAD54, Tankyrase2 was also shown to bind to MERIT40 [18]. To verify if the reported interaction takes place on the chromatin, we investigated the ability to TNKS1 to recruit MERIT40 when tethered to the lacO array chromatin. Interestingly, the colocalization frequency of TNKS1 and MERIT40 was nearly 100% suggesting a strong interaction between the two proteins (Fig 8A). Furthermore, the colocalization is independent of the PARP activity of TNKS1, as the catalytic dead mutant TNKS proved just as efficient in recruiting MERIT40 as its wild type counterpart (Fig 8A). These results are in agreement with previous results showing that MERIT40 is not a PARylation target of TNKSs and this interaction does not involve the PARP domain [18].

MERIT40 was shown to be required for the integrity of the BRCA1A complex containing RAP80, BRCA1, BRCC36 and CCDC98 [24] [25]. We thus tested if other subunits of this complex are also loaded to chromatin bound by TNKS1. We indeed found that RAP80 was recruited to the lacO array with 100% efficiency in a PARP activity independent manner by TNKS1 (Fig 8A). To verify that TNKS1 tethering does not trigger the DDR, we also observed γ-H2AX, MDC1 and 53BP1 accumulation at the TNKS1 bound array, but none of these factors or modifications could be detected (S7A Fig). To test the potential role of TNKSs in recruiting BRCA1A subunits to chromatin after DNA damage, we depleted TNKS1 and 2 in U2OS17 cells and tested the efficiency of MERIT40 and RAP80 recruitment to ISce-I induced DSBs (Fig 8B). Compared to the scrambled control, downregulation of TNKS expression decreased the association of all these factors with DSBs (Fig 8B). On the other hand, downregulation of TNKSs had no effect on 53BP1 recruitment and H2AX phosphorylation (S7B Fig). Similar results were obtained in U2OS cells treated with NCS; TNKS1/2 depletion resulted in a substantial decrease in the % of cells positive for RAP80 foci (Fig 8C), while it had no effect on the ratio of foci-positive cells for γ-H2AX, MDC1 or 53BP1 (S7C Fig). Furthermore, the proper loading of the subunits of the BRCA1A complex to pure DSBs is significantly affected when the Tankyrase binding mutant version of MDC1 is bound to the lacO array compared to the control (Fig 8D).

MERIT40 was previously reported to play a fundamental role in assuring the integrity of the BRCA1A complex. To determine whether BRCA1 loading to the array after TNKS1 tethering is mediated by MERIT40 or is due to a direct protein-protein interaction between BRCA1 and TNKSs, we depleted MERIT40 from U2OS17 cells using siRNA and tested the BRCA1 recruitment at the TNKS1 bound array. We found that siMERIT40 cells had a profound defect in BRCA1 recruitment in the absence of ISce-I break that represents the condition that BRCA1 protein is recruited to the lacO array only due to the interaction with TNKSs (Fig 8E). Depletion of MERIT40 by siRNA was verified by IF (S5D Fig).

Altogether, our observations underline the role of TNKSs in mediating the association of two BRCA1-containing complexes with DNA lesions; the BRCA1A and the CtIP-BRCA1 complex.

### TNKSs function in parallel with RNF8 mediated BRCA1A complex recruitment

**Fig 9:**
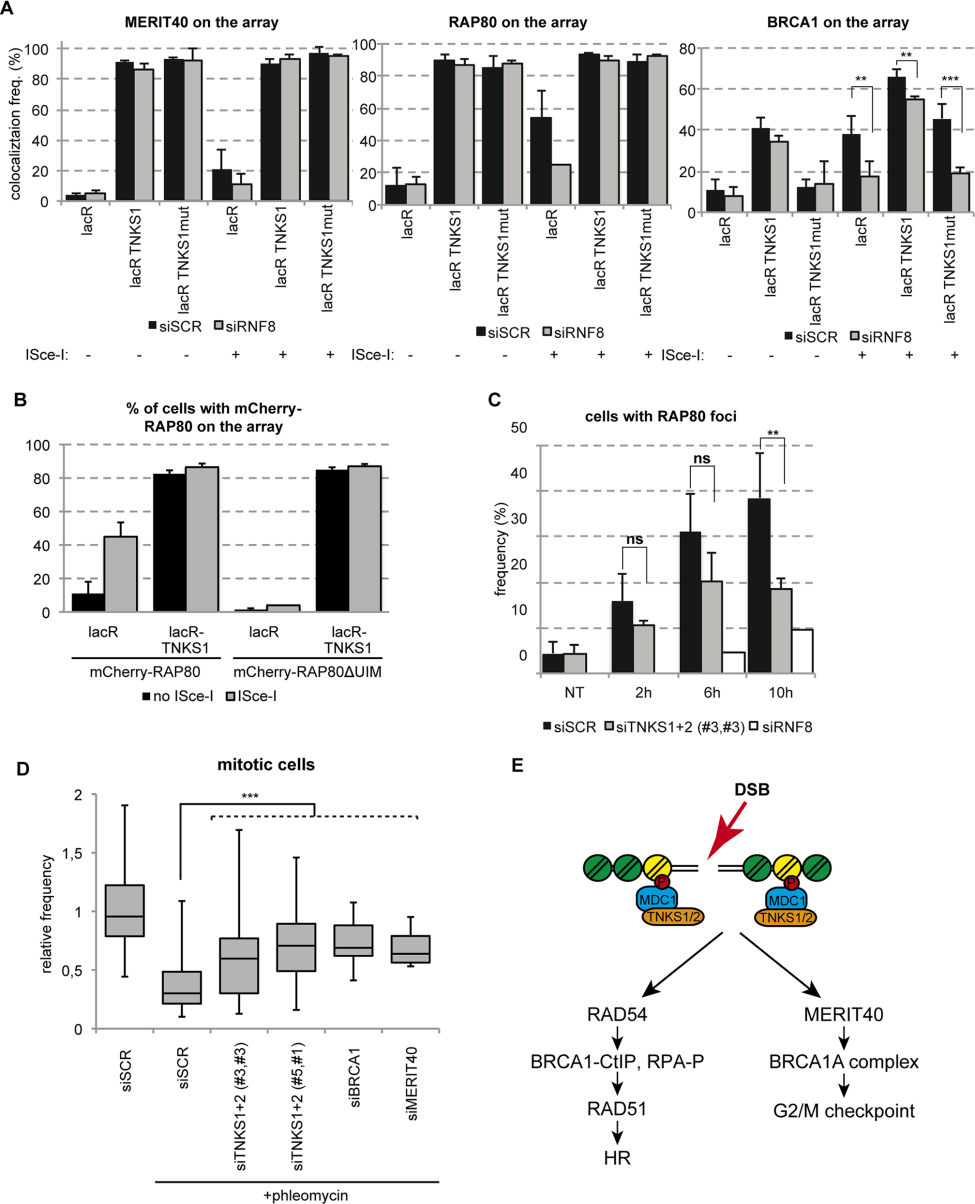
TNKSs stabilize the BRCA1A complex at DSBs and activate the G2/M checkpoint. **(A)** RNF8 mediated ubiquitination is dispensable for BRCA1A complex’s loading onto the chromatin. U2OS17 cells were transfected with siSCR control or siRNF8, and GFP-lacR, GFP-lacR-TNKS1 or its PARP activity mutant version. The frequency of MERIT40, RAP80 and BRCA1 signal on the lacO array was analyzed after immunofluorescence staining with the corresponding antibodies. Results of three independent experiments are shown with SD (N = 100). **(B)** RAP80 is recruited to TNKS1 bound chromatin in an ubiquitin-binding independent manner. U2OS17 cells were transfected with GFP-lacR or GFP-lacR-TNKS1 together with mCherry-RAP80 (wt or UIM mutant). Pure DSBs were inflicted with the cotransfection of ISce-I. Colocalization frequency of the GFP and mCherry signal is shown from three independent experiments (N = 100) with SD. **(C)** TNKS depleted cells show RAP80 foci-forming deficiency at later timepoints after DSB induction. U2OS cells were transfected with the indicated siRNAs, treated with NCS and fixed at the indicated timepoints. Results from three independent experiments are shown with SD (N = 100). **(D)** Knockdown of TNKS1/2 leads to G2/M checkpoint escape and elevated number of mitotic cells after DSB induction compared to the control. U2OS cells were transfected with the indicated siRNAs. 48 hours later cells were treated with Phleomycin and released for 6 hours. Mitotic cells were detected by an anti- histone H3 S10P fluorescence staining and their frequency determined. Values are represented on a Whisker box plot as relative to the control from three independent experiments. **(E)** Schematic action of TNKSs in DSB repair is shown.

Our data point to a TNKS dependent recruitment of BRCA1A complex to DSBs mediated by MERIT40. However, it was previously shown that RAP80 and MERIT40 recruitment to DNA lesions depends on RNF8-dependent ubiquitination [26, 27] [28]. To investigate whether the recruitment of RAP80, MERIT40 and BRCA1 by TNKSs depends on RNF8, we depleted RNF8 by siRNA and we tested their recruitment efficiency on the lacO array after TNKS1 tethering in the absence or presence of ISce-I break. We find that RNF8 depletion does not affect the recruitment of RAP80 and MERIT40 to the lacO locus upon tethering of TNKS1 (Fig 9A). At the same time siRNF8 transfected cells have only a minor reduction in the BRCA1 recruitment efficiency on tethered TNKS1 *in vivo* (Fig 9A). To further test whether the ubiquitin binding property of RAP80 is responsible for the interaction with TNKSs, we assessed the recruitment of the UIM deletion mutant RAP80 not binding to DSBs to tethered TNKS1 [29]. Interestingly, we observed that the wild type and the UIM mutant RAP80 are equally recruited to the lacO array after TNKSs tethering (Fig 9B). These findings suggest that TNKS interact with the BRCA1A complex in a ubiquitin independent manner.

To investigate the relationship of the two modes of recruitment of BRCA1A complex to breaks (the RNF8 dependent and the TNKS1 dependent), we sought to assess the kinetics of recruitment of RAP80 and MERIT40 to lesions induced by NCS upon depletion of RNF8 or TNKS1/2. As it was reported before [26, 27], RNF8 depletion affects dramatically the initial recruitment of RAP80 to breaks. On the other hand, in cells that are depleted for TNKS1 and 2, RAP80 foci formation is mainly affected at later time points, suggesting that RNF8 is essential for the recruitment of RAP80 and TNKSs for its stabilization at breaks (Fig 9C). We were unfortunately unable to detect MERIT40 foci at NCS induced breaks.

Since MERIT40 and BRCA1 were shown to be required for proper activation of the G2-M checkpoint after DNA damage [25, 30], we quantified the mitotic fraction of U2OS cells treated with phleomycin in control and TNKS depleted cells. In cells transfected with the non-targeting control siRNA there was a sharp drop in the number of cells undergoing mitosis after DNA damage induction, but in cells treated with siRNAs depleting TNKSs a significant percentage of cells could pass to the mitotic phase similarly to siMERIT40 and siBRCA1 conditions (Fig 9D).

Our data suggest that Tankyrases associate with DSBs to 1. promote homology-mediated repair by regulating resection and RAD51 loading and to 2. activate the check point, by stabilizing the BRCA1A complex (Fig 9E).

## Discussion

MDC1 is a key regulator of DDR signaling at the chromatin surrounding DSBs [13]. It has been suggested to function as a platform for the recruitment of the ATM kinase and the perpetuation of ubiquitination by mediating the binding of the ubiquitin ligases RNF8 and RNF168 to DNA lesions [13, 31]. Here we provide evidence for novel roles of MDC1. The MDC1-dependent Tankyrase recruitment to DSBs reported here plays considerable roles in DSB repair by HR and in retention of the BRCA1A complex at lesions (Fig 9E).

Tankyrase 1 is a multifunctional poly(ADP-ribose) polymerase that was first shown to localize to telomeres through its interaction with the shelterin component TRF1 [32, 33]. A recent study also showed that TNKS1 depletion by shRNA leads to DNA damage response activation exemplified by increased γ-H2AX and 53BP1 foci [33]. It has been proposed that DNA damage is caused by persistent cohesion of telomeric sequences after the completion of S-G2 and during mitosis [33]. In *C*. *elegans*, Tankyrase expression was shown to increases after DNA damage [34].

Our study provides evidence for a direct role of TNKSs in Double Strand Break Repair (DSBR) through their binding to MDC1 that can offer an additional explanation for the persistent DNA breaks observed in the absence of TNKS1. We describe here different modes of action for TNKSs in DSBR. First we found that TNKSs affect Homologous Recombination by controlling DNA end resection and therefore the recruitment of RAD51. Our data show that TNKS1 binding to chromatin is sufficient to promote binding of BRCA1 and CtIP and thereby promote resection as it was shown before [35].

Then we found that TNKSs load the BRCA1A complex to DSBs. MERIT40 is a regulator of BRCA1A complex integrity and its depletion destabilizes the complex and affects its recruitment to DSBs [24, 25, 30]. In agreement with this, we observe that TNKSs mediate the loading of MERIT40, RAP80 and BRCA1 to chromatin. RNF8-dependent ubiquitination was shown to be important for MERIT40 and RAP80 recruitment to DNA lesions [26] [28] [24]. In this study, we demonstrate a novel mechanism for the recruitment of the BRCA1A complex to DSBs that involves interaction of MERIT40 with TNKSs. Our observations show that ubiquitination acts as the primary signal for the recruitment of the BRCA1A components and TNKSs are needed for the stabilization of the complex at the sites of damage. Moreover, our data clearly show that the stabilization and the interaction with TNKSs are independent of ubiquitin binding.

The BRCA1A complex was reported to play an inhibitory role in resection and depletion of some of its subunits leads to increased Homologous recombination efficiency [36, 37]. Our experiments show that TNKSs, although interact with the BRCA1A complex, regulate HR in a positive way. This can be explained by the fact that TNKSs promote the recruitment of two BRCA1 containing complexes to lesions, the BRCA1-CtIP and the BRCA1A complexes.

Several recent reports highlighted the importance of poly(ADP)ribosylation in modulating the function of BRCA1 containing complexes. TNKSs were described to form a complex with ATM and BRCA1 during mitosis that assures proper mitotic progression through regulation of the poly(ADP)ribosylation of NuMA1 [38]. Also, poly(ADP)ribosylation was shown to be important for the rapid recruitment of the BRCA1-BARD1 heterodimer to DSBs [39]. Furthermore, PARylation of BRCA1 was recently reported to play a role in stabilizing the RAP80-BRCA1 interaction and modulating its DNA binding capacity [40].

As Tankyrase substrates were shown to get degraded [18], an appealing hypothesis would be that MDC1 *per se* is PARylated and in a timely controlled manner gets erased from the chromatin by subsequent proteasomal degradation. In support of this idea, MDC1 is degraded upon DNA damage in an RNF4 dependent manner [20, 41]. Also, in a recent study the *Drosophila* homologue of Tankyrase was reported to have a direct role in activating the 26S proteasome subunit’s activity through PARylating its partner, PI31 [42]. Moreover, RNF146, a ubiquitin ligase that is reported to ubiquitinate proteins PARylated by TNKSs, was also reported to play a role in DNA repair [43]. Although this is a very exciting possibility that could provide evidence as to how DDR is switching off, our efforts to detect MDC1 PARylation by TNKS1 *in vitro*, or stabilization of MDC1 protein in cells in which TNKSs are downregulated, were unsuccessful. Nevertheless, we cannot exclude the possibility that our experimental conditions were not optimal to detect such a modification.

An interesting feature of the above- described MDC1-TNKS interaction is that it is controlled by MDC1 itself. Indeed, we observed that in the absence of the MDC1 “PST” domain there is a substantial increase in the percentage of cells that display TNKS and tethered MDC1 colocalization, suggesting that the “PST” domain has an inhibitory effect on the interaction. Our finding that TNKS recruitment to DSBs occurs mainly during the S-G2 phases indicates that the “PST” domain may undergo post-translational modification(s) during cell cycle that induce conformational changes in MDC1 modulating its binding to TNKSs. Notably, the MDC1 “PST” domain was previously shown to get phosphorylated after DNA damage [44, 45] and MDC1 is also phosphorylated during mitosis, further supporting our hypothesis that a “PST”-dependent structural change might occur [46]. Moreover, we showed that TNKS PARP activity is not required for the association with DSBs, which is in agreement with our yeast two-hybrid data showing that the TNKS ankyrin repeat domain interacts with MDC1. The ankyrin repeat domain was previously shown to be important for the majority of interactions between TNKSs and their known binding partners [33].

The requirement for two TBDs is another interesting feature of the MDC1-TNKSs interaction. Both TBDs are highly conserved in MDC1 sequences from different species, highlighting their importance in MDC1 functions (S8 Fig). It is noteworthy, that although in the context of the FL MDC1 the two TBDs have an additive role in the recruitment of TNKSs to chromatin, the “interII” domain including TBD1 is more proficient in loading TNKSs to the lacO array upon tethering. Of note, TBD2 lies in the hinge region between the two BRCTs and TNKS binding might thus be further regulated by the interaction of MDC1 with γ-H2AX. Future structural studies might highlight the importance of the two TBDs.

Recently the idea of targeting Tankyrases in cancer treatment has emerged due to the discovery of their role in Wnt signalling [47]. Moreover, TNKS depletion was shown to be selectively lethal in a BRCA1 deficient background through a mechanism that was proposed to involve supernumerary chromosomes [48]. However, in the light of our new data about TNKS’s role in G2-M checkpoint activation, escape from the checkpoint might result in elevated DNA damage that can be also exemplified by supernumerary chromosomes [48]. Therefore, our results open a new perspective on targeting Tankyrases for cancer therapy.

## Materials and Methods

### Cell culture

U2OS, U2OS17 and U2OS19 cells were cultured at 37°C in DMEM supplemented with 4.5g/l glucose, 10% FCS and gentamicin. GCV6 cells and HRind cells were cultured as previously described [17, 49].

### Chemicals were purchased from the following suppliers

JetPEI (PolyPlus), Fugene6 (Promega), Lipofectamine 2000 (Invitrogen), INTERFERin (PolyPlus), Neocarzinostatin (NCS, Sigma) Phleomycin (Sigma), XAV-939 (Tocris), Mimosine (Sigma), RO-3306 (Millipore), ATMi KU55933 (Tocris).

### Antibodies

The following antibodies were used during our study:

Anti-lacR (gift from Dr A. Belmont), anti-flag (F7425, Sigma), anti-flag M2 (F3165, Sigma), anti-Myc (clone 9E10), anti- γ-H2AX (ab22551, abcam), anti-H2AX (ab11175, abcam) anti-53BP1 (NB100-304, Novus), anti-RAD51 (PC130, Calbiochem), anti-BRCA1 (OP92 and OP93, Calbiochem), anti-MERIT40 (gift from Dr J. Chen), anti-histone H3 S10P (51TA2H12, Active Motif), anti-MDC1 (MDC1-50, Sigma), anti-TNKS (ab13587, abcam), anti-HA (11867423001, Roche), anti-ATM (Novus NB100-104), anti-DNA-PKcs (Millipore 04–1024), anti-tubulin (Sigma-Aldrich T5168), anti-Merit40 (A302-516A Bethyl), anti RAP80 (A300-763A Bethyl), anti-CtIP (Clone 14–1 Active motif), anti-phospho-RPA32 S4/8 (A300-245A, Bethyl).

Secondary antibodies for IF were purchased from Life Technologies, secondary antibodies for WB were from Jackson Immuno Research.

The home-made rabbit polyclonal anti-MDC1 antibodies (clones #2991 and #2992) were developed using the ETDAEEGTSLTASVVADVRK peptide corresponding to the 577–597 aa region of MDC1 as antigen. The peptide was coupled to ovalbumine and used to immunize two rabbits.

### siRNAs

All siRNAs used in the study were purchased from Dharmacon. Except if not precised, they were “On-Target plus SMARTpool” siRNAs containing four different sequences against the target mRNA.

siSCR: D-001810-10-20
siMDC1: M-003506-04
siATM: L-003201-00
siMERIT40: L-020702-01
siBRCA1: D-003461-06
siRNF8: L-006900-00

Custom designed siRNAs were the following:

siMDC1_UTR: CGGCUUGACCUCUGUGAUAUU
siTNKS1#3 (D-004740-03) GGAAGUAGCUGAAUAUCUU
siTNKS1#5 (J-004740-05) CUACAACAGAGUUCGAAUA
siTNKS2#1 (D-004741-01) GGAAAGACGUAGUUGAAUAUU
siTNKS2#3 (D-004741-03) AGACAGAUCUUGUUACAUU
siTNKS2_UTR: GGAAAUAUGCUGUCAGUUUUU

### Plasmids

The FN-TNKS1 and MN-TNKS2 expressing plasmids in pCDNA 3.1 backbone having a flag or myc tag and NLS signal were gifts from Dr S. Smith. The PARP catalytic dead TNKS1 was subcloned together with its flag tag and the NLS from a retroviral vector (gift from S. Smith) to pCDNA 3.1 using the XhoI and HindIII sites. The flag-TNKS2 expressing plasmid and its mutant version were purchased from AddGene. The GFP-lacR-TNKS1 expressing plasmid and its mutant version were cloned in two steps: first the GFP cassette was added to the HindIII site of the FN-TNKS1 plasmid, and then the lacR coding sequence was subsequently cloned to the NotI site. mCherry-lacR-MDC1 deletion constructs were the results of amplifying by PCR the appropriate size fragment and subcloning it to the mCherry-lacR vector [21]. HA-ISce-I was a gift from Maria Jasin. The cDNA of RAP80 wt and UIM deletion mutant was amplified by PCR using the CFP-RAP80wt and CFP-RAP80ΔUIM vectors [50] and was subsequently cloned to the mCherrry-C2 vector. Directed mutagenesis to obtain the TBD mutant versions of MDC1 was done using the QuikChange site-directed mutagenesis kit. The siRNA resistant form of TNKS1 was generated by directed mutagenesis using the same kit. The mutations of the two Tankyrases rendering them catalytically inactive are:

TNKS1: H1184A and E1291A (described in [51])
TNKS2: E1138A (described in [18])

### Immunoprecipitation and WB

HEK293T cells were transfected with the indicated plasmids using Fugene 6 (Promega) following the supplier’s recommendations. Two days later cells were scraped in TENT buffer (25 mM Tris-HCl 7.5, 2 mM EDTA 150 mM NaCl, 1% Triton X-100 protease inhibitor cocktail, (Roche)) and incubated in ice for 15 min. The extracts were centrifuged and the supernatant was pre-cleared and subsequently incubated with the anti-Flag-M2 antibody for 2 hours. Protein G sepharose beads were added to the antibody-protein complex for 16 hrs. The beads were washed 3 times with TENT buffer and the bound fraction was eluted in SDS loading buffer. For the endogenous MDC1 IP, HEK293T cells were treated or not with 100 ng/ml NCS for 15 min and released for 1 hour prior to cell lysis and Immunoprecipitation procedure as described above.

1% input or the immunoprecipitated fractions were separated on SDS PAGE and transferred to Nitrocellulose membrane. The membrane was blocked in 5% milk PBS and incubated O/N at 4°C with the indicated antibodies. After three washes HRP-conjugated secondary antibodies were added for 1 hour and the membrane was developed using ECL detection kit (GE Healthcare).

### Fractionation

U2OS cells -treated or not with NCS- were incubated in Hypotonic buffer (10 mM Hepes-KOH pH 7.9, 1.5 mM MgCl_2_, 10 mM KCl, 0.5 mM DDT) on ice for 15 min followed by douncing using the loose pestle. After centrifugation of the samples the supernatant was considered the cytoplasmic fraction and the pellet (nuclei fraction) was further lyzed using RIPA buffer. The protein content of the cytoplasmic and nuclear fractions was determined by Bradford assay (BioRad) and equal protein amounts were analyzed by SDS-PAGE.

### FACS

FACS analysis of GFP (for HR efficiency) or propidium iodide staining (for cell cycle analysis) was conducted on a FACS Calibur (Becton Dickinson) and the results were quantified using FlowJo.

### RNA purification and RT-qPCR

RNA was isolated from the cells using TriReagent (Molecular Research Center) as suggested by the manufacturer. RT-qPCR reactions were done in triplicates using the QuantiTect SYBR Green PCR Kit (Qiagen) in a Roche Light Cycler. All the expression values were normalized to that of CyclophilinB.

HR assay was described earlier [17, 49].

### Immunofluorescence staining

Staining was done following standard IF protocol. Briefly, cells were washed with PBS, fixed in 4% PFA and permeabilized with PBS 0.1% tritonX-100. Blocking and incubation in antibodies were performed in 10% heat inactivated FCS, washes were done with PBS 0.1% triton X-100. Nuclei were counterstained with DAPI and cells were mounted using the ProLong Gold antifade reagent of Molecular Probes. Confocal microscopy pictures were taken at a Leica SP2 microscope, Z stack width was usually 0.5μm. For foci positive cell counting, at least 100 cells were analyzed for each condition. For RAD51 staining a cell was considered positive bearing >3 foci. For BRCA1, RAP80 and MDC1 staining a cell was considered positive bearing >5 foci.

### Generation of lesions by multiphoton laser

Multiphoton laser striping was conducted on a Leica SP5 system, using a DMI6000 microscope with a 63x Plan Apo 1.4 objective and a Coherent Chameleon laser at 800nm wavelength. The microscope stage was equipped with a 37°C Life Imaging System box. For inflicting DNA breaks in several cells in a mosaic, the Taile-Scan option of the Leica program was applied.

### Mitotic cell counting

U2OS cells were seeded in 6well plates and transfected by the indicated siRNAs as suggested by the supplier, using Lipofectamine2000 (Invitrogen). Twenty-four hours later cells were trypsinized and seeded at 5000cell/well concentration in glass-bottom, black 96 well plates. Another 24 hours later, cells were treated with 20μg/ml Phleomycin for 1 hour and released for 6 hours. Cells were fixed and processed for IF with an anti-histone H3 S10P antibody to mark mitotic cells. The percentage of positive cells was determined by the automated InCell1000 analyzer.

### Yeast two-hybrid analysis

Yeast two-hybrid screening was performed by Hybrigenics Services, S.A.S., Paris, France (http://www.hybrigenics-services.com).

The coding sequence for human MDC1 aa 742–1698 (GenBank accession number gi: 132626687) was PCR-amplified and cloned into pB66 as a C-terminal fusion to Gal4 DNA-binding domain (N-Gal4-MDC1-C). The construct was checked by sequencing and used as a bait to screen a highly complex random-primed human placenta cDNA library constructed into pP6. pB66 and pP6 derive from the original pAS2ΔΔ and pGADGH plasmids, respectively. 90 million clones (10-fold the complexity of the library) were screened using a mating approach with Y187 (matα) and CG1945 (mata) yeast strains as previously described [52]. A total of 169 colonies were selected on a medium lacking tryptophan, leucine and histidine. The prey fragments of the positive clones were amplified by PCR and sequenced at their 5’ and 3’ junctions. The resulting sequences were used to identify the corresponding interacting proteins in the GenBank database (NCBI) using a fully automated procedure. A confidence score (PBS, for Predicted Biological Score) was attributed to each interaction as previously described [53].

### Treatments for cell cycle blocking

Cells were blocked in G1 by a 20-hour treatment of 0,5mM mimosine, released from this block for 5 hours for S phase, or blocked for 20 hours by 10uM RO-3306 to enrich the population in G2.

### Counting % of cells with colocalizing TNKS and γ-H2AX

Immunofluorescence staining of FN-TNKS1 and γ-H2AX was analyzed by microscopy. Cells were considered positive for colocalization after inspection of the merged images. Quantification was done in 50 cells for each condition. Intensity plots were generated by the Image J software.

## Supporting Information

S1 Fig Results of the yeast two-hybrid screen. (DOCX)

S2 Fig The PARP activity of TNKS1 is not required for its recruitment to pure DSBs in vivo. U2OS17 cells were transfected with cherry-lacR, FN-TNKS1mut and ISce-I (bottom line pictures). Immunofluorescence staining was performed on the cells, representative pictures are shown with the merged picture. For quantification of the colocalizing signal, see Fig 4B.
(TIF)

S3 Fig (A) Cellular distribution of TNKS was analyzed in asynchronous U2OS cells (DMSO) or cell populations enriched in G1 (mimosine), S (mimosine+release) or G2 (RO) phases of the cell by franctionation and Western blotting. TNKSs are present both in the cytoplasm (cp) and in the nuclear fraction (nucl). Their expression level is higher is S/G2 phases of the cell cycle and TNKS1 accumulates slightly more in the nucleus. (B) FACS analysis of cell populations from panel A. (C) Tankyrase knock-down doesn’t change the cell cycle profile of U2OS cells significantly. Cells were transfected with the indicated siRNAs and harvested for propidium iodide staining forty-eight hours later. Cell cycle state of cells was determined by FACS analysis. Results of two independent experiments are shown with SEM. (D) TNKS depletion has no effect on the portion of replicating cells. U2OS cells were transfected with the indicated siRNAs and pulse-labelled with EdU for 1hour. Cells were stained with the Click-iT EdU imaging kit as suggested by the supplier and the number of positive cells was determined (marked on the right).
(TIF)

S4 Fig Depletion of TNKSs has no effect on the recruitment of MDC1 to DSBs in vivo. (A) U2OS17 cells were transfected with the indicated siRNAs and ISce-I, and immunofluorescence staining was performed against MDC1. Values were obtained in three independent experiments (N = 100). (B) U2OS cells were transfected with the indicated siRNAs and treated with NCS. % of cells harboring γ-H2AX foci was determined, relative values compared to the control are shown.
(TIF)

S5 Fig Verification of knock-down efficiencies of siRNAs against (A) TNKS1 and TNKS2, (B) MDC1, (C) BRCA1,(D) MERIT40. (TIF)

S6 Fig (A) The Tankyrase binding domains of MDC1 are essential for efficient RAD51 foci formation. U2OS cells were transfected with the indicated siRNA and either a plasmid expressing lacR, or a plasmid expressing lacR-MDC1 (wild type or TBD mutant). Cells were treated with NCS and fixed 6 hours later. Immunofluorescence staining against RAD51 was performed and cells with more than 5 foci were quantified. (B) U2OS cells were transfected and treated as on panel (A). Representative images of RAD51 and MDC1 pattern are shown. (C) Tankyrase inhibition doesn’t affect HR efficiency. Cells that have been pretreated with 3μM XAV-939 (TNKSi) for 24 hours have no detectable defect in the repair pathways compared to the control. (D) XAV-939 stabilizes both TNKS1 and 2 proteins in the cells.
(TIF)

S7 Fig (A) Tethering of TNKS doesn’t induce DDR. U2OS17 cells were transfected with GFP-lacR, GFP-lacR-TNKS1 or GFP-lac- TNKS1mut. Twenty four hours after transfection cells were fixed and immunostained for g-Η2ΑΧ 53BP1 or MDC1. Percent of cells harboring positive signal on the lacO array was determined. Results of three independent experiments are shown with SEM (N = 100). (B) Depletion for TNKSs does not affect the early DDR at pure DSBs in vivo. U2OS17 cells were transfected with the indicated siRNAs and DSB was induced with transfecting the ISce-I endonuclease. The frequency of cells harboring positive signal on the array was determined as on panel (A). (C) TNKS depletion doesn’t affect foci formation of g-Η2ΑΧ 53BP1 or MDC1. U2OS cells were transfected with the indicated siRNAs and treated with NCS 48 hours later. Cells were fixed and the number of foci-positive cells determined in three independent experiments. Results are represented as relative to the control with SEM (N = 100).
(TIF)

S8 Fig Multiple alignment of predicted TBD sequences found in the MDC1 sequence of different organisms. The consensus sequence and amino acids corresponding to it are marked in red.
(TIF)
