# Supplementary material for: Tankyrases Promote Homologous Recombination and Check Point Activation in Response to DSBs
Source: PLoS Genet. 2016 Feb 4;12(2):e1005791. doi: 10.1371/journal.pgen.1005791 (PMC4741384; doi:10.1371/journal.pgen.1005791)
Supplement: S1 Fig — (DOCX) [file pgen.1005791.s001.docx]

**Bait : human MDC1 (1-2089)**

**Prey library : human placenta_RP4**

| **Gene name** | **Start…Stop (nt)** | **Frame** | **InFrame STOP** | **PBS** |
| --- | --- | --- | --- | --- |
| TOPBP1 | -22…895 | IF |  | D |
| NEDD9 | 2466…3746 | IF | x | D |

**Bait : human MDC1 (742-1698)**

**Prey library : human placenta_RP4**

| **Gene name** | **Start…Stop (nt)** | **Frame** | **InFrame STOP** | **PBS** |
| --- | --- | --- | --- | --- |
| COPS5 | 96…894 | IF |  | F |
|  | 144…881 | IF |  | F |
|  | 135…1208 | IF | x | F |
| CSH1 | -46…553 | IF |  | E |
|  | -36…584 | OOF |  | E |
|  | 133…503 | OOF |  | E |
| EEF1A | -49…558 | IF | x | C |
|  | -42…554 | OOF | x | C |
| FLJ53539 | 168… | IF |  | D |
| HEY-L | 33…646 | IF |  | F |
| NPIPL3 | -181…536 | IF | x | D |
| PLEKHH1 | 156…1119 | IF |  | E |
| POLR2E | -19…719 | IF | x | D |
| PSG8 | 129…1145 | IF |  | D |
| RANBP9 | 372…1468 | IF |  | F |
| SH3BP2 | 387…806 | IF |  | D |
| SH3BP4 | 2841…4508 | IF | x | D |
| **TNKS1** | 912…2058 | IF |  | D |
|  | 912… | IF |  | D |
| **TNKS2** | 135…2455 | IF |  | E |

| Global PBS (for interactions represented in the screen) | |
| --- | --- |
| A | Very high confidence in the interaction |
| B | High confidence in the interaction |
| C | Good confidence in the interaction |
| D | Moderate confidence in the interaction. This category is the most difficult to interpret because it mixes two classes of interactions :  -false positif interactions  -interactions hardly detectable by the Y2H technique |
| E | Interactions involving highly connected prey domains, warning of non-specific interaction  -prey proteins that are known to be highly connected due to their biological functions  -proteins with a prey interacting domain that contains a known protein interaction motif or a biochemically promiscous motif |
| F | Experimentally proven technical artifacts |

**Supplementary Figure 1.**

Results of the Yeast two-hybrid screen.
