# Supplementary figures and images for: Tankyrases Promote Homologous Recombination and Check Point Activation in Response to DSBs
Source: PLoS Genet. 2016 Feb 4;12(2):e1005791. doi: 10.1371/journal.pgen.1005791 (PMC4741384; doi:10.1371/journal.pgen.1005791)

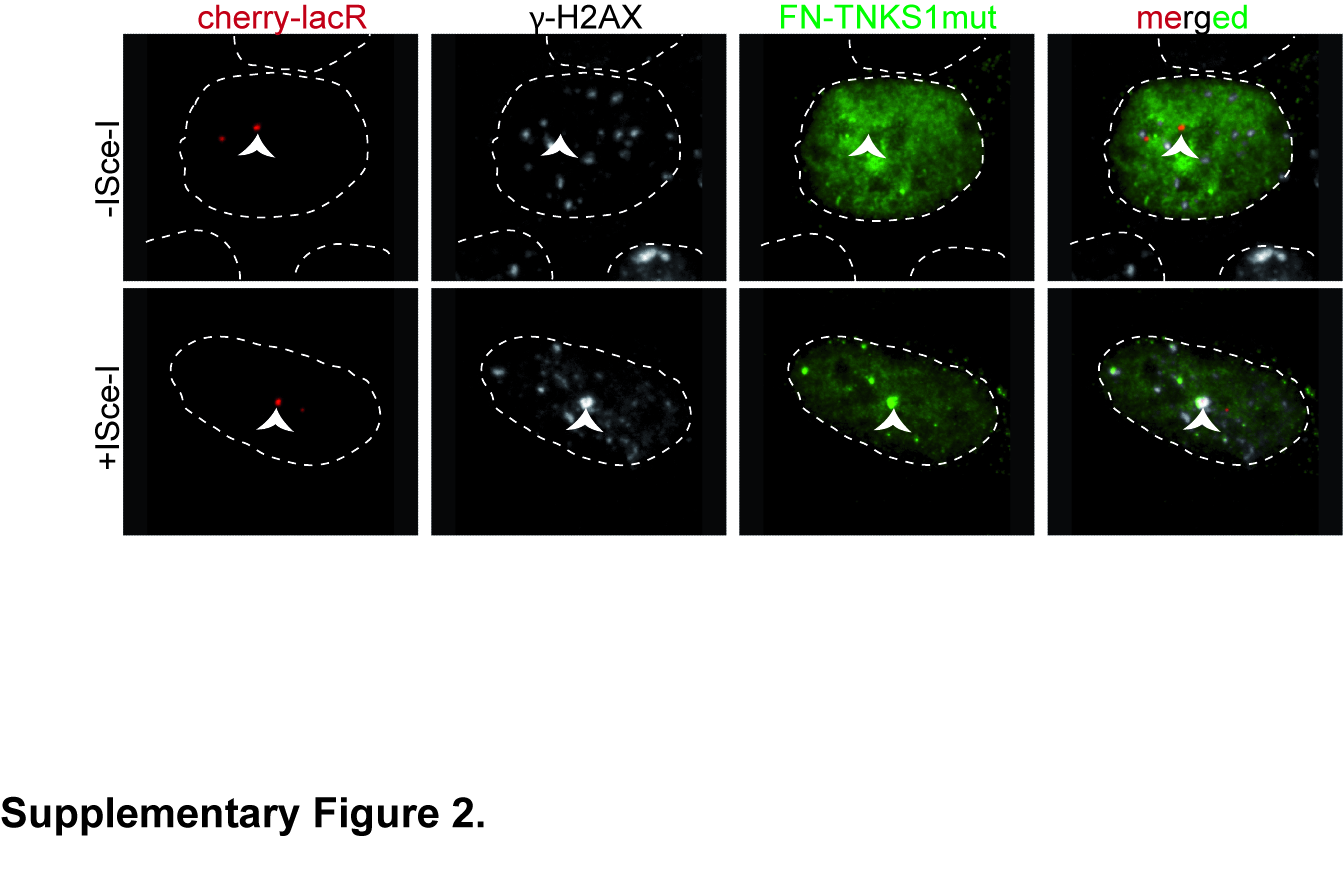

Supplement: S2 Fig — U2OS17 cells were transfected with cherry-lacR, FN-TNKS1mut and ISce-I (bottom line pictures). Immunofluorescence staining was performed on the cells, representative pictures are shown with the merged picture. For quantification of the colocalizing signal, see Fig 4B. (TIF) [file pgen.1005791.s002.tif]

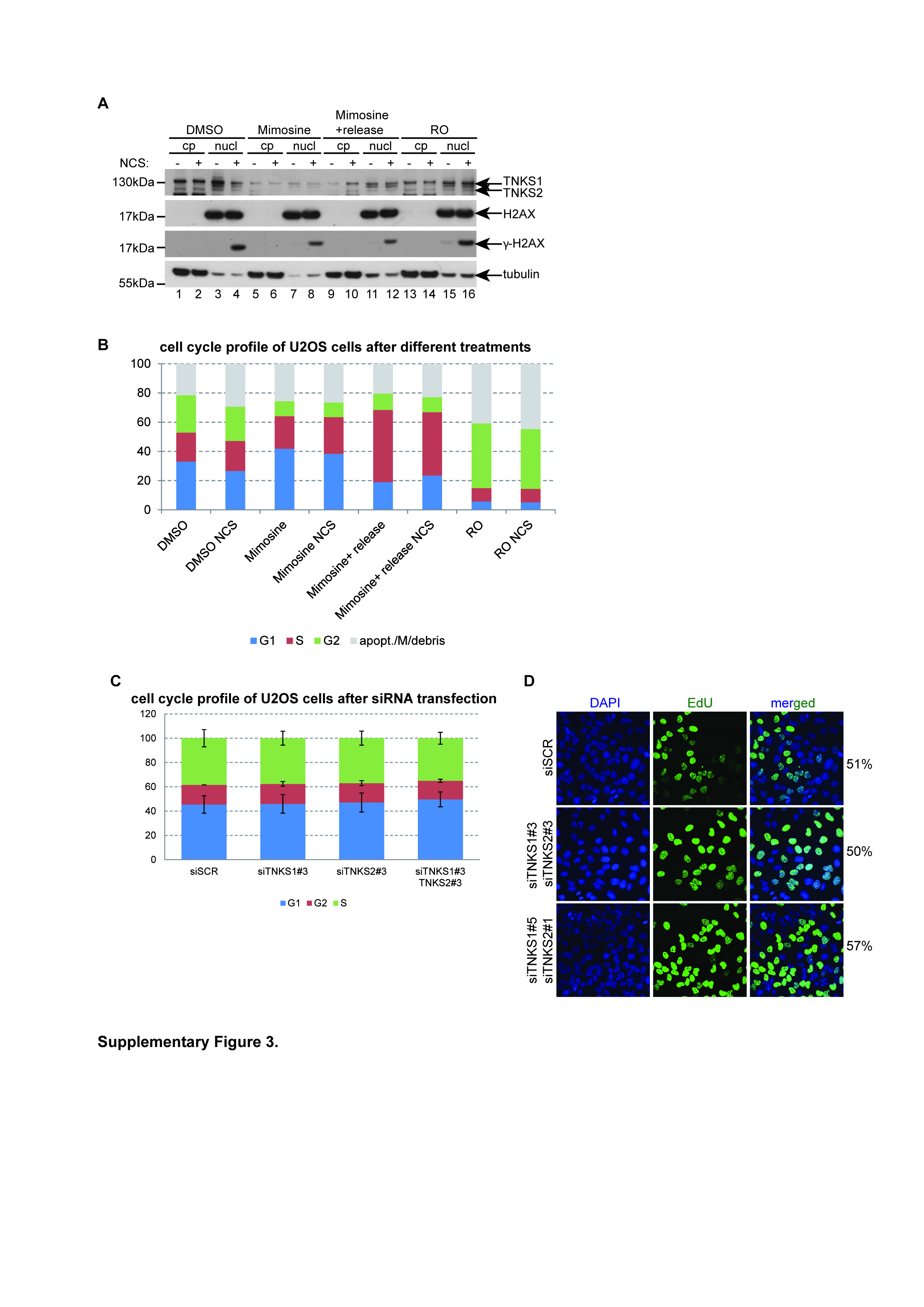

Supplement: S3 Fig — TNKSs are present both in the cytoplasm (cp) and in the nuclear fraction (nucl). Their expression level is higher is S/G2 phases of the cell cycle and TNKS1 accumulates slightly more in the nucleus. (B) FACS analysis of cell populations from panel A. (C) Tankyrase knock-down doesn’t change the cell cycle profile of U2OS cells significantly. Cells were transfected with the indicated siRNAs and harvested for propidium iodide staining forty-eight hours later. Cell cycle state of cells was determined by FACS analysis. Results of two independent experiments are shown with SEM. (D) TNKS depletion has no effect on the portion of replicating cells. U2OS cells were transfected with the indicated siRNAs and pulse-labelled with EdU for 1hour. Cells were stained with the Click-iT EdU imaging kit as suggested by the supplier and the number of positive cells was determined (marked on the right). (TIF) [file pgen.1005791.s003.tif]

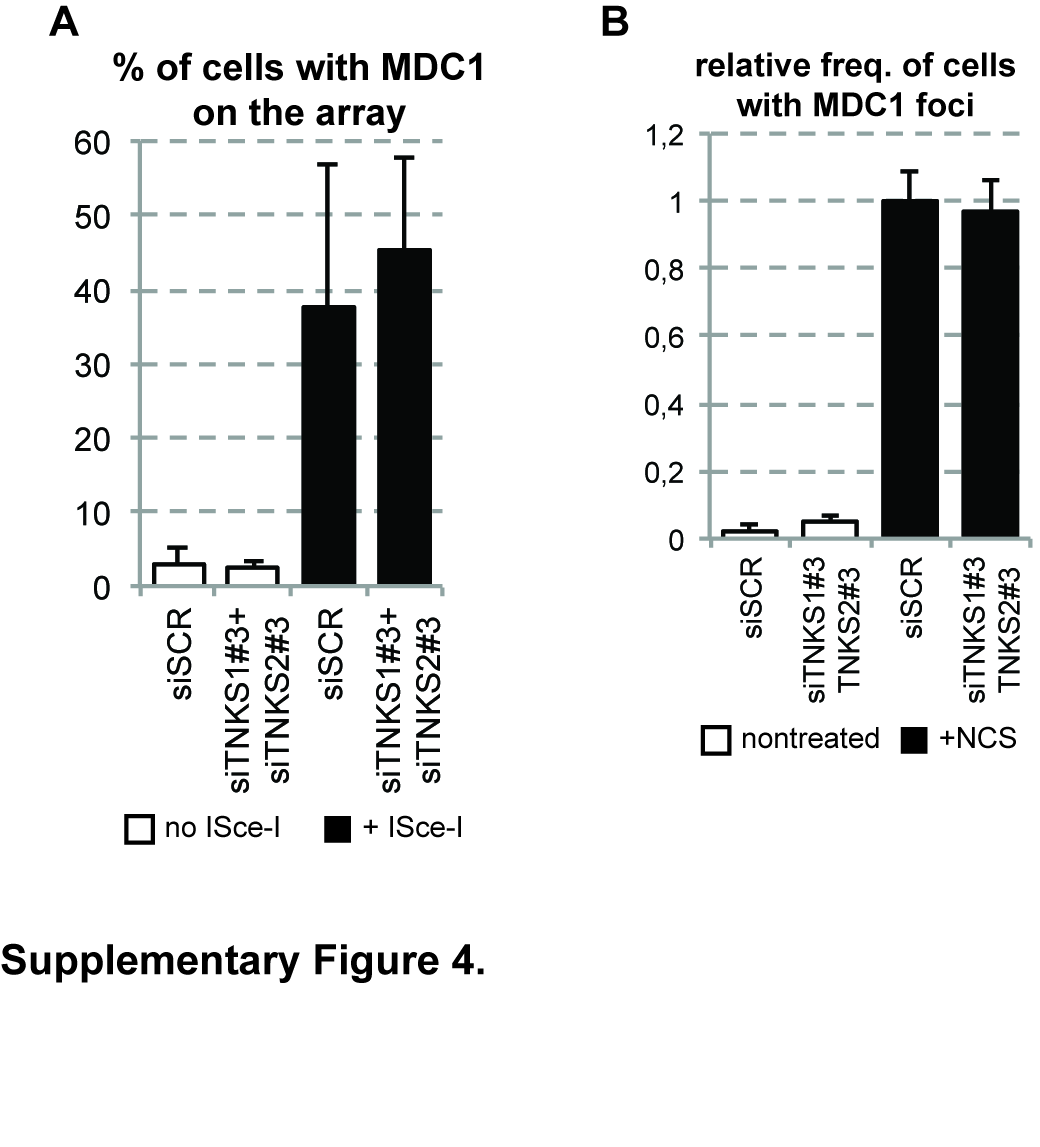

Supplement: S4 Fig — (A) U2OS17 cells were transfected with the indicated siRNAs and ISce-I, and immunofluorescence staining was performed against MDC1. Values were obtained in three independent experiments (N = 100). (B) U2OS cells were transfected with the indicated siRNAs and treated with NCS. % of cells harboring γ-H2AX foci was determined, relative values compared to the control are shown. (TIF) [file pgen.1005791.s004.tif]

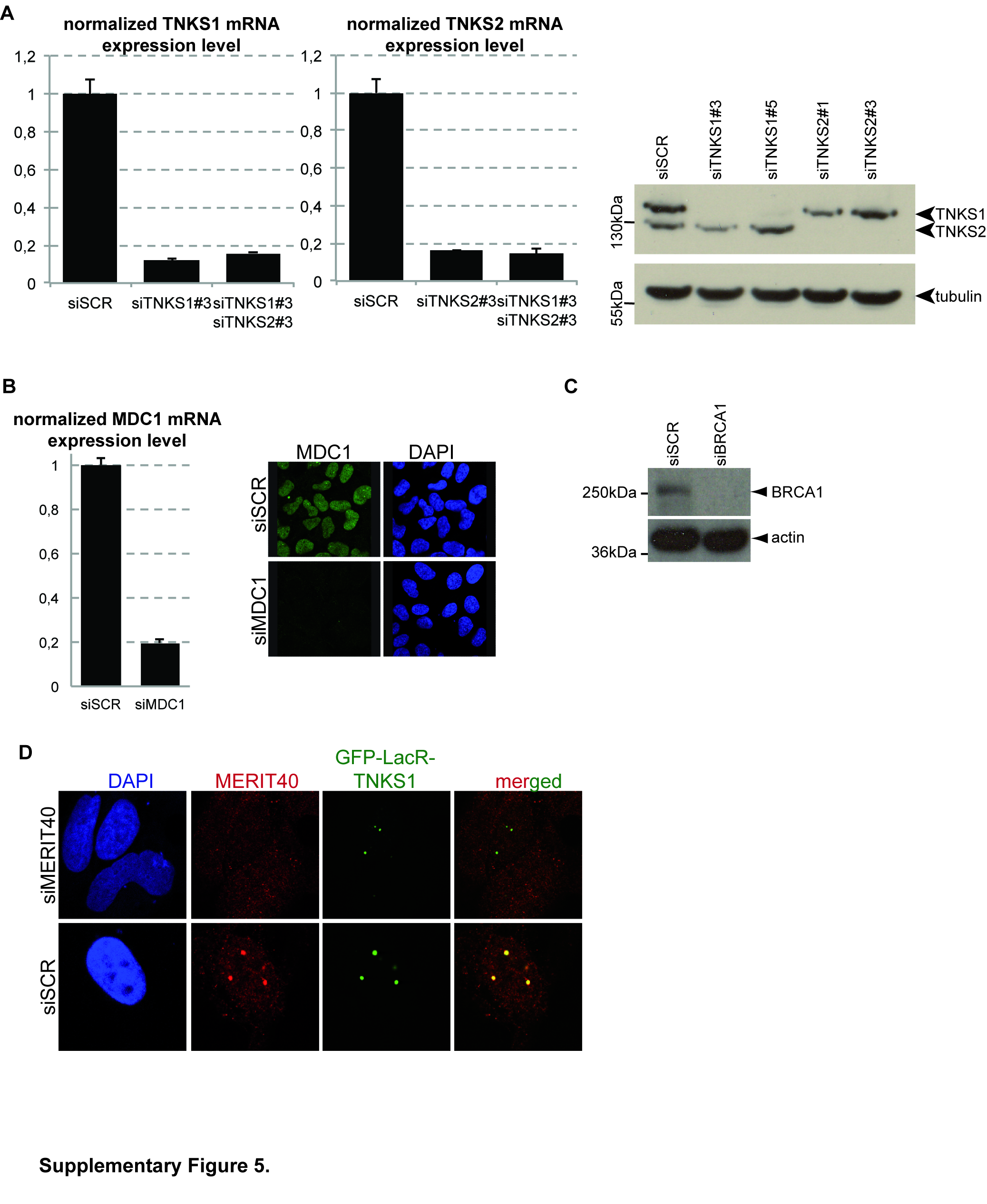

Supplement: S5 Fig — (TIF) [file pgen.1005791.s005.tif]

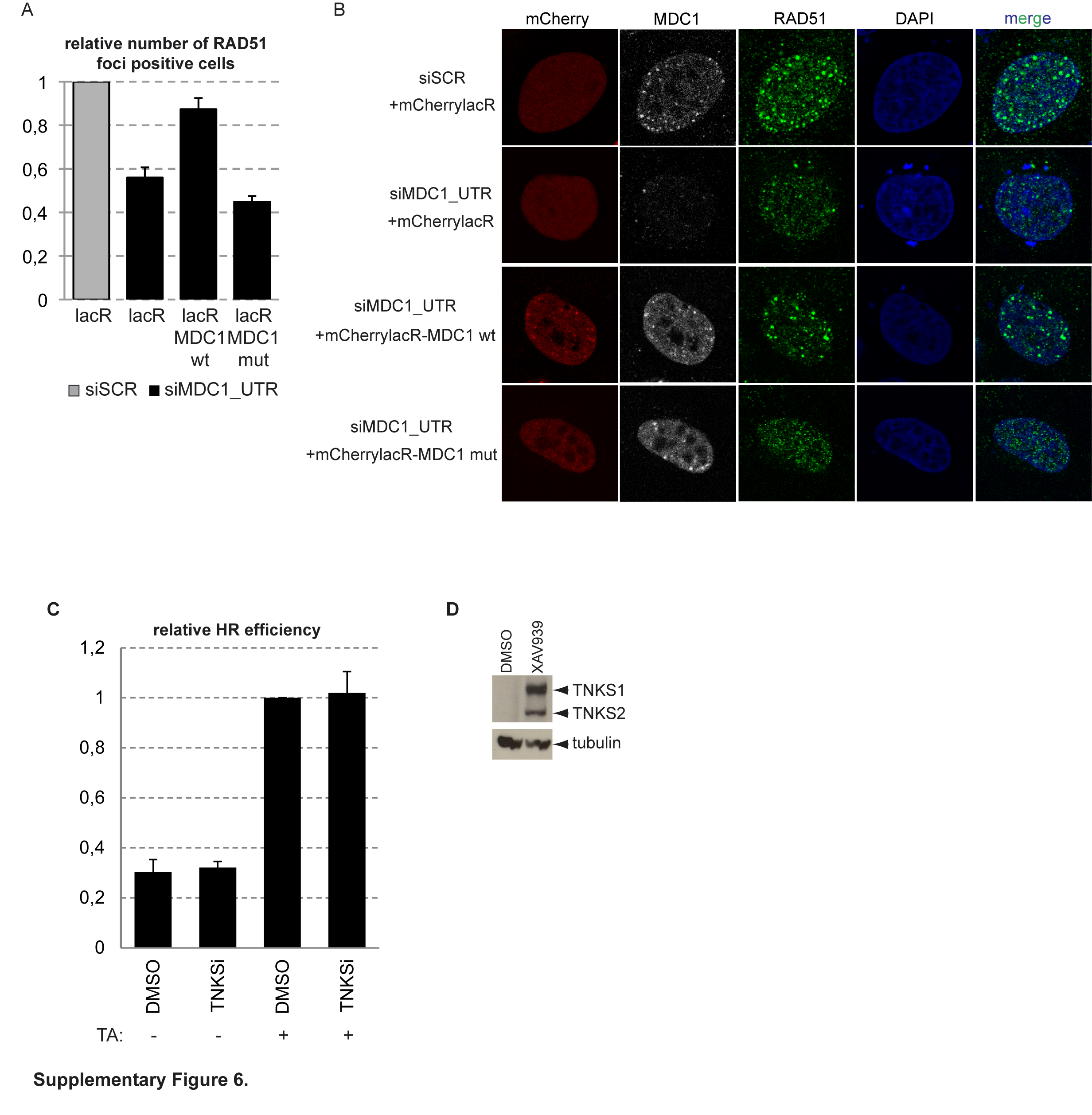

Supplement: S6 Fig — U2OS cells were transfected with the indicated siRNA and either a plasmid expressing lacR, or a plasmid expressing lacR-MDC1 (wild type or TBD mutant). Cells were treated with NCS and fixed 6 hours later. Immunofluorescence staining against RAD51 was performed and cells with more than 5 foci were quantified. (B) U2OS cells were transfected and treated as on panel (A). Representative images of RAD51 and MDC1 pattern are shown. (C) Tankyrase inhibition doesn’t affect HR efficiency. Cells that have been pretreated with 3μM XAV-939 (TNKSi) for 24 hours have no detectable defect in the repair pathways compared to the control. (D) XAV-939 stabilizes both TNKS1 and 2 proteins in the cells. (TIF) [file pgen.1005791.s006.tif]

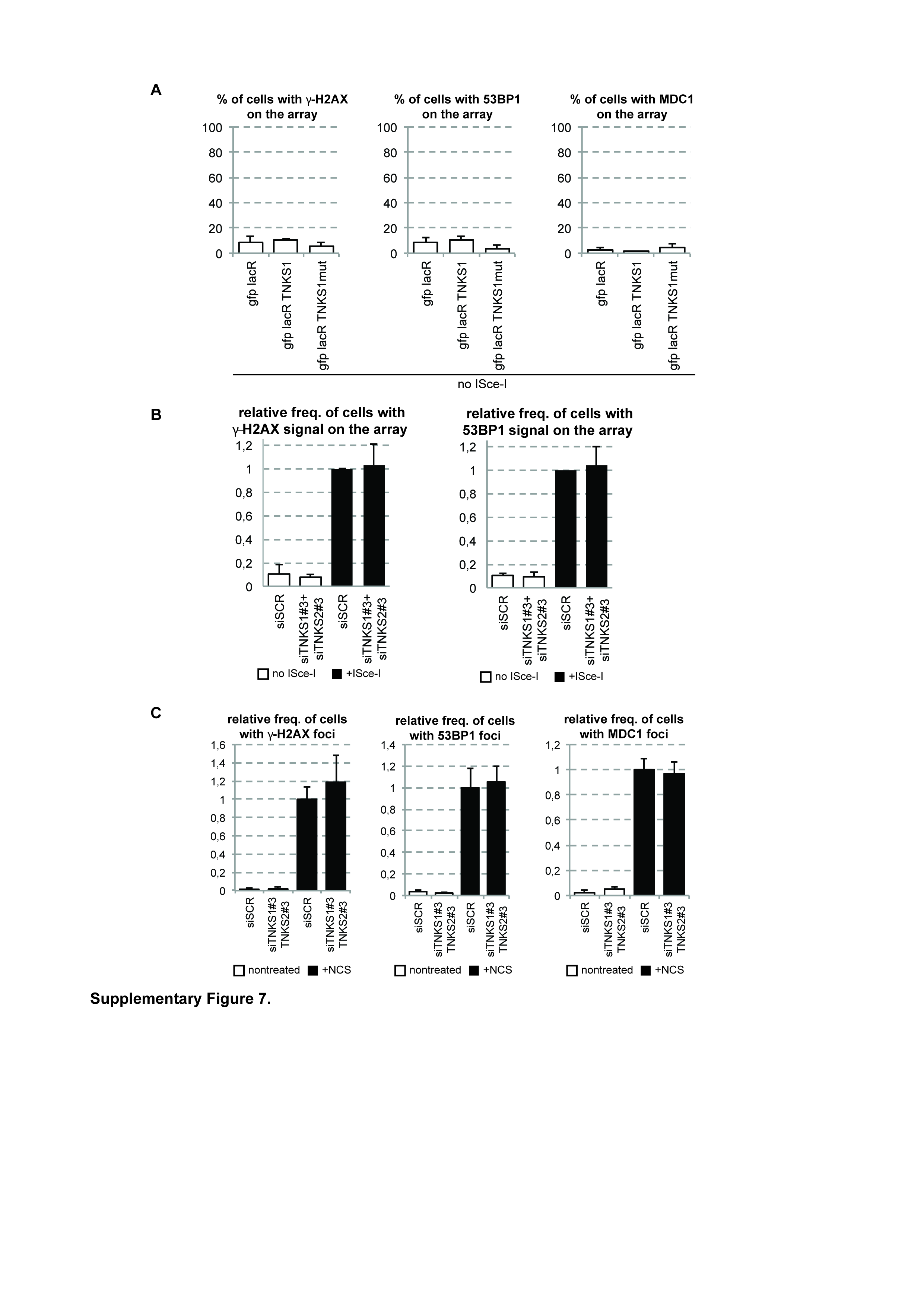

Supplement: S7 Fig — U2OS17 cells were transfected with GFP-lacR, GFP-lacR-TNKS1 or GFP-lac- TNKS1mut. Twenty four hours after transfection cells were fixed and immunostained for g-Η2ΑΧ 53BP1 or MDC1. Percent of cells harboring positive signal on the lacO array was determined. Results of three independent experiments are shown with SEM (N = 100). (B) Depletion for TNKSs does not affect the early DDR at pure DSBs in vivo. U2OS17 cells were transfected with the indicated siRNAs and DSB was induced with transfecting the ISce-I endonuclease. The frequency of cells harboring positive signal on the array was determined as on panel (A). (C) TNKS depletion doesn’t affect foci formation of g-Η2ΑΧ 53BP1 or MDC1. U2OS cells were transfected with the indicated siRNAs and treated with NCS 48 hours later. Cells were fixed and the number of foci-positive cells determined in three independent experiments. Results are represented as relative to the control with SEM (N = 100). (TIF) [file pgen.1005791.s007.tif]

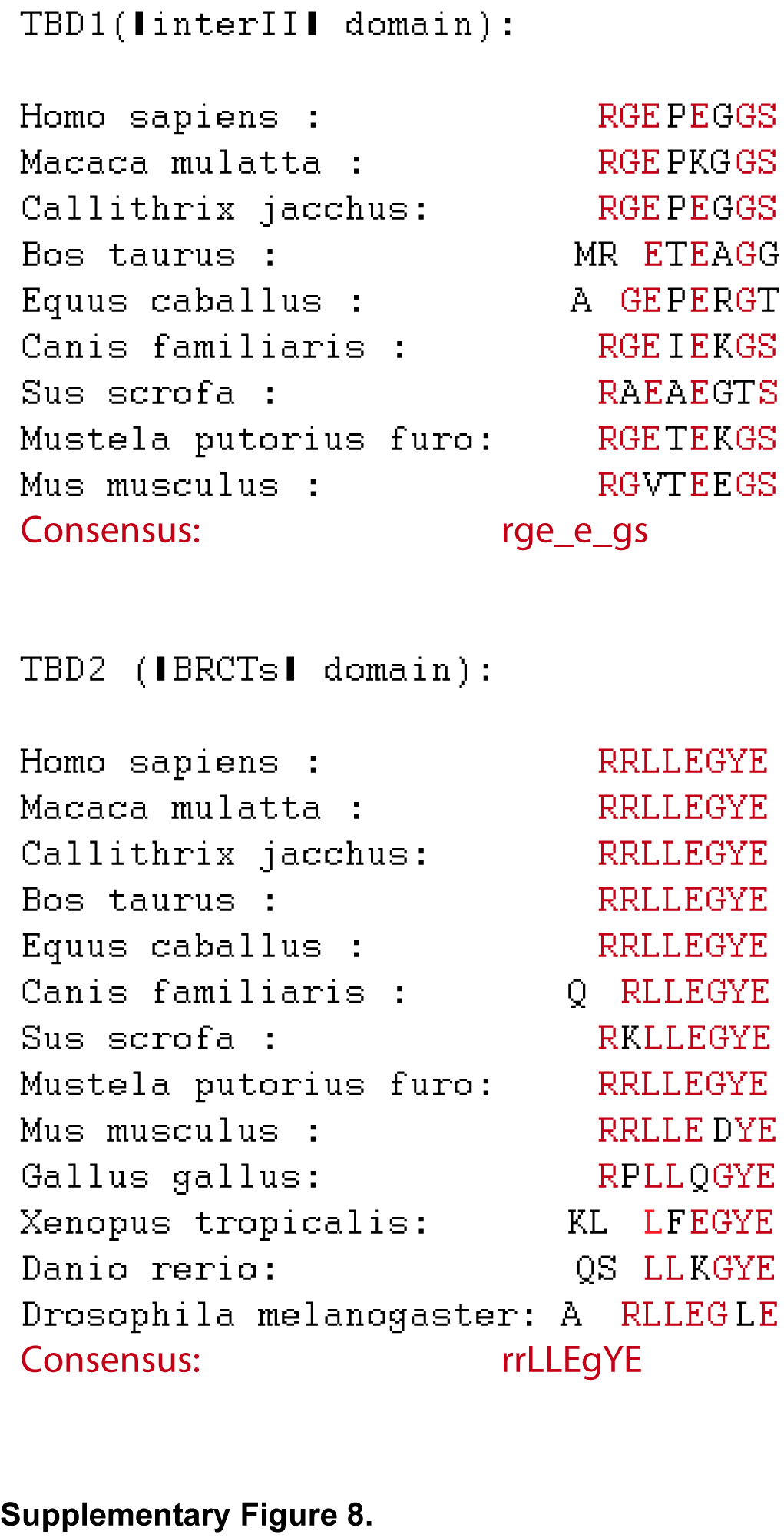

Supplement: S8 Fig — The consensus sequence and amino acids corresponding to it are marked in red. (TIF) [file pgen.1005791.s008.tif]
